# Comparative analysis of the transcriptome during single-seed formation of *Castanea henryi*: regulation of starch metabolism and endogenous hormones

**DOI:** 10.1186/s12870-023-04102-4

**Published:** 2023-02-13

**Authors:** Qi Qiu, Xiaoming Tian, Guolong Wu, Juntao Wu, Xiaoming Fan, Deyi Yuan

**Affiliations:** 1 Key Laboratory of Cultivation and Protection for Non-Wood Forest Trees, Ministry of Education, Central South University of Forestry and Technology, Changsha, 410004 China; 2 Key Lab of Non-Wood Forest Products of State Forestry Administration, Central South University of Forestry and Technology, Changsha, 410004 China; 3 Hunan Botanical Garden, Changsha, 410116 China

**Keywords:** *Castanea henryi*, Fertile ovule, Abortive ovule, Starch, hormone, Transcriptome analysis

## Abstract

**Background:**

In seed plants, the ovule is the precursor to the seed. The process of ovule development and differentiation is regulated by multiple factors, including starch metabolism and endogenous hormones. *Castanea henryi* produces nuts with high nutritional value. However, the high proportion of empty buds restricts the commercial use of the tree. Previous studies have shown that the empty bud phenotype is closely related to ovule abortion. If none of the ovules in the ovary expand rapidly and develop in 7–8 weeks after pollination, an empty bud will form. Therefore, we studied the development and molecular mechanisms underlying single seed formation in *C. henryi*.

**Results:**

We found that 49 days after pollination (DAP) is a critical period for the formation of fertile and abortive ovules. The morphology and starch distribution of the fertile and abortive ovules differed significantly at 49 DAP. The fertile ovules were smooth and round in appearance, with a large amount of starch. In contrast, abortive ovules were smaller with only a small amount of starch. The embryo sac of the abortive ovule proceeded to develop abnormally, and the entire ovule lacked starch. We identified 37 candidate genes involved in metabolism with potential roles in the regulation of starch levels. Three ADP-glucose pyrophosphorylase (AGPase) genes, one granule-bound starch synthase (GBSS) gene, and two beta-amylase genes could affect starch accumulation. The levels of auxin, cytokinins, gibberellins, and jasmonic acid in fertile ovules were higher than those in abortive ovules. In addition, the levels of endogenous abscisic acid and salicylic acid in abortive ovules were higher than those in fertile ovules of the same age, consistent with the expression patterns of genes related to the synthesis of abscisic and salicylic acid and signal transduction. We identified and mapped the differentially expressed genes associated with hormone synthesis and signal transduction.

**Conclusions:**

These results improve our general understanding of the molecular mechanisms underlying single seed development in *C. henryi* and the phenomenon of empty buds, providing directions for future research.

**Supplementary Information:**

The online version contains supplementary material available at 10.1186/s12870-023-04102-4.

## Background

Ovules carry the female gametophyte, which develops into a seed after fertilization. This is indispensable for the formation of seeds and the normal growth of angiosperms [1]. In addition to being consumed by humans, seeds also have a high economic value, as they are used for the production of animal feed and raw chemical materials, and the research and development of medicine [2]. Crop yield often depends on the quantity and quality of the seeds, while the quality of each seed depends largely on the number, size, and proper development of the ovules [3]. Although each ovule has the potential to be fertilized until it forms viable embryos, obstacles arising during sexual reproduction can prevent some ovules from developing. The occurrence of ovule abortion may be affected by physiological, biochemical, embryological, environmental, and molecular factors, including male sterility [4], female sterility [5], poor pollination or fertilization [6], temperature [7], and water [8]. Consequently, understanding the mechanisms of ovule development is of great importance from both scientific and economic perspectives. The ovule development process is regulated by several factors, including starch metabolism and endogenous hormone regulation. Starch actively participates in the metabolic processes of embryonic development and regulates the dynamic balance of carbohydrate metabolism in plants. Moreover, starch is the most significant means of carbohydrate storage and therefore the primary energy store in some seeds, particularly in *Castanea*.

Starch metabolism is an essential part of seed development, and has a direct impact on grain yield and quality. Starch metabolism in plants is aided by a variety of complex and coordinated mechanisms governed by several enzymes [9, 10]. The key processes and pathways involved in starch metabolism in higher plants include starch synthesis and degradation. In storage tissues, sucrose is hydrolyzed into monomeric sugars by invertase (InVS) and sucrose synthase (SUSY) to form uridine diphosphate glucose (UDP-glucose) and fructose [11]. Subsequently, these products are transformed into glucose-1-phosphate (G-1-P) by UDP-glucose pyrophosphorylase. G-1-P forms adenosine diphosphate glucose (ADPG) through the action of ADP-glucose pyrophosphorylase (AGPase). ADPG is a direct precursor of starch synthesis and synthesizes amylose under the action of ADP-Glc pyrophosphorylase (AGPase) and granule-bound starch synthase (GBSS) [11, 12]. ADPG synergizes with AGPase, soluble starch synthase (SS), SBE (starch branching enzyme), and the starch debranching enzyme (DBE) to synthesize amylopectin. Synthesized starch is mainly degraded by alpha-amylases (AMY), beta-amylases, or through the phosphorylation pathway [13]. AMY hydrolyzes the alpha-(l,4) glycosidic bonds of amylose and amylopectin in the substrate molecule [14]. Beta-amylase cleaves α-(1,4) glycosidic bonds from the non-reducing ends of polysaccharides to generate maltose [15].

Endogenous hormone regulation also plays an important role in embryonic development [16]. Among them, auxins play an important role in the formation of female gametophytes and sporophyte organization. l-Tryptophan aminotransferase of Arabidopsis1(*TAA1*) encodes a key protein involved in auxin synthesis, which is under-expressed in developmentally deficient ovules [17]. Cytokinins (CKs) negatively regulate cell proliferation in the sporophyte tissue surrounding the developing female gametophyte. Therefore, a lack of CKs leads to an increase in the number of cells in the ovule, which in turn leads to an increase in seed size [18]. Gibberellins (GAs) regulate ovule development. In a previous study, treating flowers with an exogenous GA spray significantly increased the proportion of sweet cherries with nacreous ovules, prolonged the embryo sac life span, and increased the seed setting rate [19]. Abscisic acid (ABA) plays an important role in embryonic development, promoting the synthesis of storage proteins during seed maturity; consequently, a sharp increase in ABA is an important factor leading to embryo abortion [20]. Jasmonic acid (JA) regulates ovule senescence, and the JA signal response factor *PbMYC2* can directly activate the transcription of senescence-related genes, thereby inducing the death of pear ovules. Salicylic acid (SA) also contributes to ovule abortion [21]. These studies demonstrate the synergistic effect of multiple hormones in regulating embryonic abortion in plant species.

*Castanea henryi* is a tall deciduous tree of the Fagaceae family. The nuts of *C. henryi* have a smooth and attractive appearance, and their sweet taste and nutritional value are making them increasingly popular among consumers [22, 23]. The production of *C. henryi* has become a pillar industry allowing farmers to increase their income in certain mountainous areas of China. However, the high rate of empty chestnut buds leads to low production and reduced income [24]. Considering the increasing demand for chestnuts in China, studying the key factors affecting the formation of empty buds and finding a way to artificially increase chestnut yield are urgent problems to be solved. Previous studies have shown that the rate of empty bud production is closely related to ovule abortion [25, 26]. Currently, research focusing on the development of *C. henryi* ovules is limited, and there have been no studies on macromolecule dynamics during single seed formation. In this study, we studied the external morphology of ovule development, starch distribution, metabolic enzyme activity, and endogenous hormone levels during the development of fertile and abortive ovules. Candidate genes related with starch biosynthesis metabolism and hormone synthesis were identified by sequencing the transcriptomes of selected developmental stages.

## Results

### Microscopic observation on the formation of fertile and abortive ovules

**Fig. 1:**
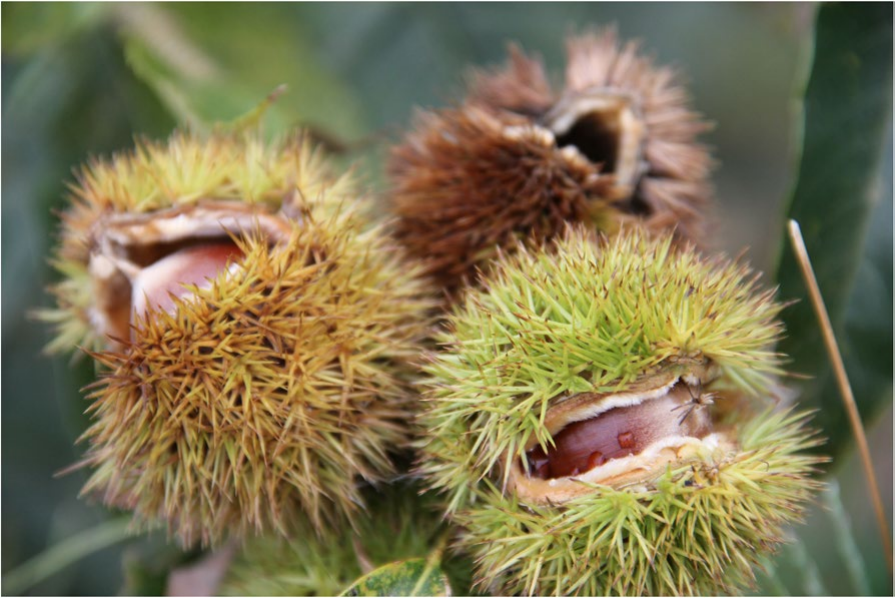
Representative picture of single seed fruiting of *C. henryi*

**Fig. 2:**
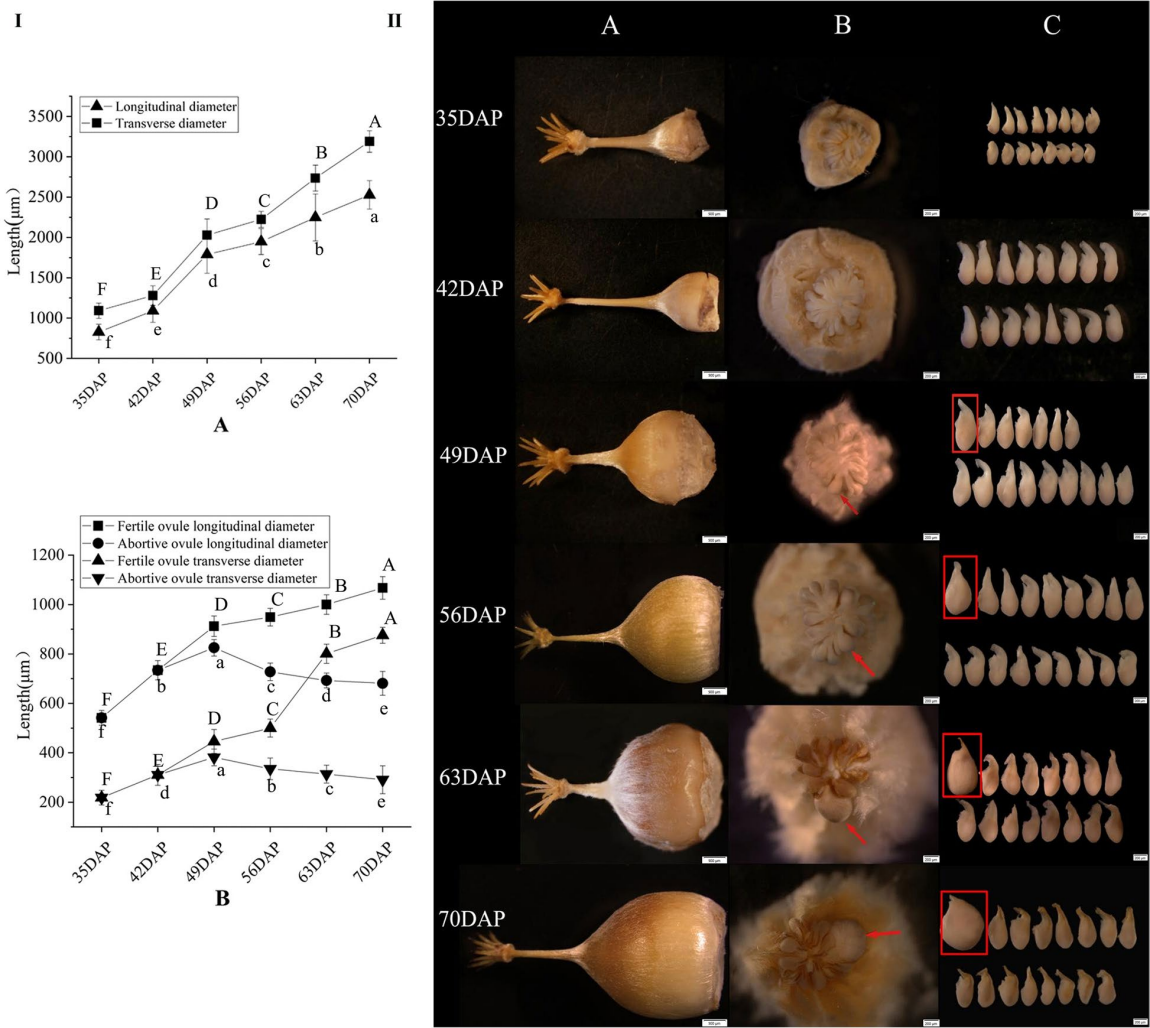
**I** The size of the *C. henryi* ovary and ovules at different developmental stages. (A) Changes in the longitudinal and transverse diameter of the ovary of *C. henryi*. (B) Comparing transverse and longitudinal diameter lengths of fertile and abortive ovules at six developmental stages. DAP: days after pollination. The values represent the mean ± the standard deviation (*n* = 3). Different lowercase letters indicate significant differences between the means (*P* < 0.05). The figure shows the analysis of the significant differences within the group. Significant differences in fertile ovules are marked with capital letters above the dotted line, and differences in aborted ovules are marked with lowercase letters below the line. **II** Developmental process of a *C. henryi* ovule between 35–70 DAP. A-C represent the ovary, interior of the ovary, and ovule, respectively

*Castanea henryi* has a compound pistil with a multi-carpel ovary and congruent style. The ovaries are wrapped with closed or semi-closed thorn buds. The ovules are all born at the ventral suture of the ovarian wall and are surrounded by many placental hairs. There are many ovules in one ovary. However, no matter what way of pollination (self pollination, outcrossing and natural pollination), only one seed will be produced (Fig. 1). The transverse diameter of the *C. henryi* ovary was always larger than the longitudinal diameter, and the growth rate of the transverse diameter was also higher than that of the longitudinal diameter at 35–70 days after pollination (DAP). The ovary diameter had a growth peak at 42–49 DAP, and the growth rate suddenly slowed at 49–56 DAP (Fig. 2I). In light of the ovary anatomy, there were usually 16–24 ovules in each ovary. Except for one fertile ovule that could develop normally, the rest of the ovules were aborted (Fig. 2II). At the outset, the size of each ovule in the same ovary was relatively consistent with no significant difference. At 49 DAP, it can be seen that one ovule being slightly larger than other ovules is the fertile ovule. The mean transverse and longitudinal diameters of the aborted ovules reached their maximum values at 49 DAP (Fig. 2I). Subsequently, the fertile ovules gradually become rounded in shape and milky white in color, full, and plump. In contrast, the abortive ovules begin to shrink and shrivel at the micropylar end, have a yellowish brown and gradually extend downward; indicating that 49 DAP describes a critical period for the formation of fertile and abortive ovules (Fig. 2II).

### Observation of starch distribution in ovule during single seed formation

**Fig. 3:**
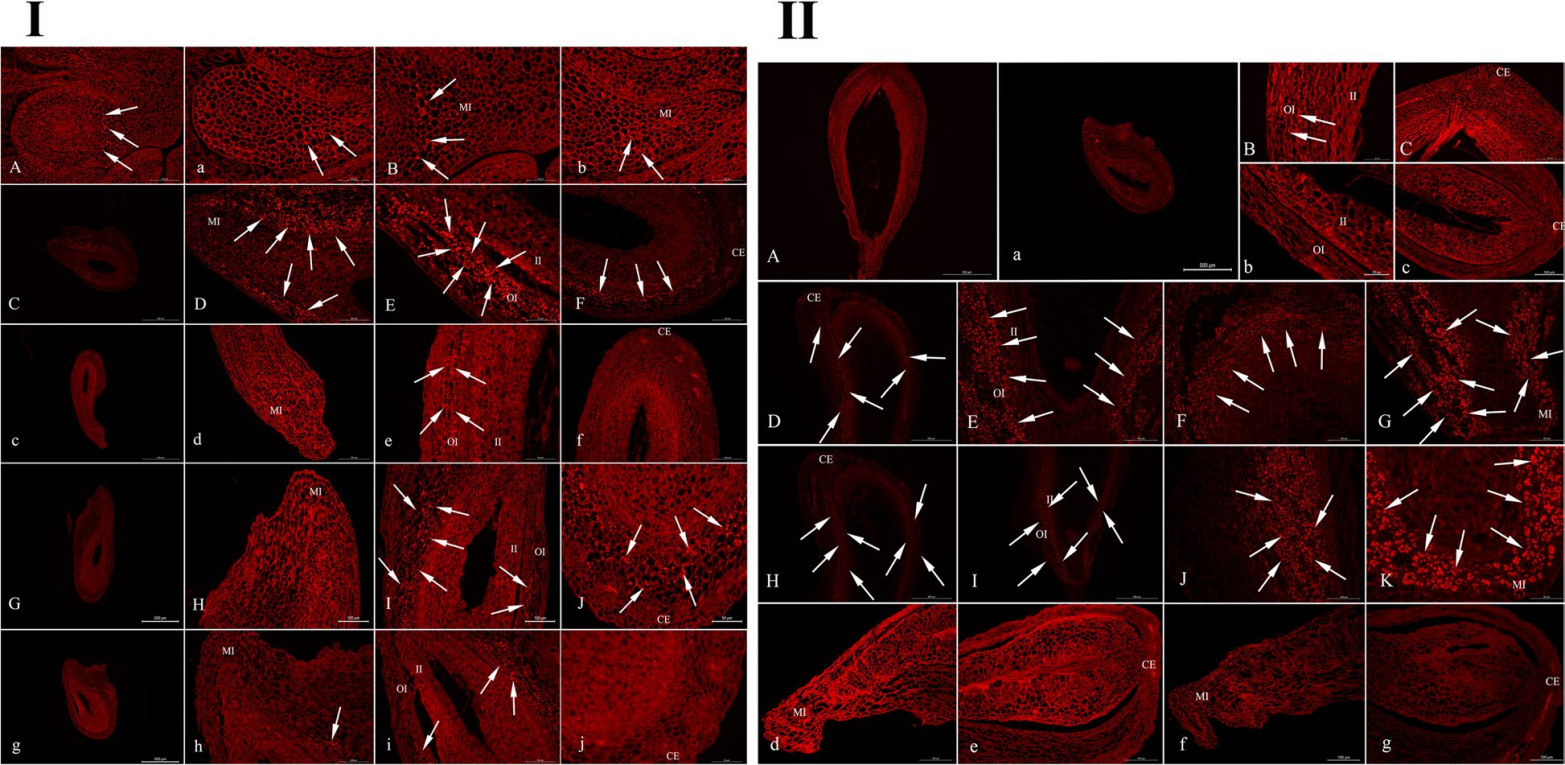
**I** Slices of starch distribution in 35 DAP, 42 DAP and 49 DAP ovules in *C. henryi*. A and B show the whole 35 DAP fertile ovule, and micropyle end and surrounding cells, respectively; a and b show the whole 35 DAP abortive ovule, and micropyle end and surrounding cells, respectively. C-F represent the whole 42 DAP fertile ovule, micropyle and surrounding cells, right and left sides of the inner and outer integuments, and chalazal end of the fertile ovule; c-f represent the whole 42 DAP abortive ovule, micropyle end and surrounding cells, right sides of inner and outer integument, and chalazal end of the abortive ovule, respectively. G-J represent the whole 49 DAP fertile ovule, micropyle end, two sides of the integument and its surrounding cells, and chalazal end, respectively; g-j represent the whole 49 DAP abortive ovule, micropyle end, both sides of integument and its surrounding cells, and the chalazal end, respectively. White arrows indicate starch grains. **II** Slices of starch distribution in 56 DAP, 63 DAP, and 70 DAP ovules in *C. henryi*. A-C represent the whole, chalazal end, and left side of integument of 56 DAP fertile ovule; a-c represent the whole, chalazal end of 56 DAP abortive ovule, and left side of the bead quilt, respectively. D-G show the chalazal end of the fertile ovule 63 DAP, spherical embryo and its cells, right side of the integument, and micropylar end of the fertile ovule; d-e show the whole 63 DAP abortive ovule. H–K show the chalazal end, 70 DAP fertile ovule, heart-shaped embryo, and both sides of the integument; f-g show the whole 70 DAP abortive ovule. II: inner integument; OI: outer integument; MI: micropyle; CE: chalazal end

To investigate the changes in starch metabolism during single-seed formation, we observed the distribution of starch. Although fertile and infertile ovules cannot be distinguished by assessing the external morphology at 35–49 DAP, they can be classified based on the paraffin section. The starch distribution in fertile ovules washigher than that in abortive ovules at 35–70 DAP. The starch distribution in fertile ovules showed an increasing trend, with a sudden decrease at 49–56 DAP and a rapid increase at 56–70 DAP (Table 1). Initially, starch grains were mainly distributed in the inner and outer integuments and chalazal ends of the fertile ovules. With the continuous development of the ovule, starch grains were greatly reduced, with only sporadic starch distribution on both sides of the fertile ovule integument (Fig. 3I). At 63–70 DAP, starch distribution greatly increased. The starch grains were almost entirely distributed over the fertile ovule, with a tight distribution and large starch grains (Fig. 3II).

**Table 1:** The changes of amount of starch grains in two types of ovules over time

Type of ovule	35 DAP	42 DAP	49 DAP	56 DAP	63 DAP	70 DAP
Fertile ovule	+ + +	+ + + +	+ +	+	+ + + + +	+ + + + + + +
Abortive ovule	+ +	+ + +	+	-	-	-

+ Indicates starch grains estimated amount unit; - indicates no satrch grains.

In aborted ovules, the starch distribution was greatest at 42 DAP and then gradually decreased until no starch distribution was observed (Table 1). First, starch grains were mainly distributed in the outer integument and chalazal end of the aborted ovules, while a small amount of starch was distributed in the inner integument. This was followed by the gradual decrease of starch distribution in the aborted ovules (Fig. 3I). Although, the abortive ovule develops normally, the embryo sac fails to do so and shows a long and narrow gap structure, and the entire ovule is completely free of starch grains (Fig. 3II).

Based on these results, three representative developmental stages (35 DAP, 49 DAP, 63 DAP) were selected for the comparative analysis of transcriptome data to better understand the starch metabolism molecules and adjustive mechanisms of ovules during the single-seed setting of *C. henryi*.

### Changes in the contents of endogenous hormones during the formation of single seeds

**Fig. 4:**
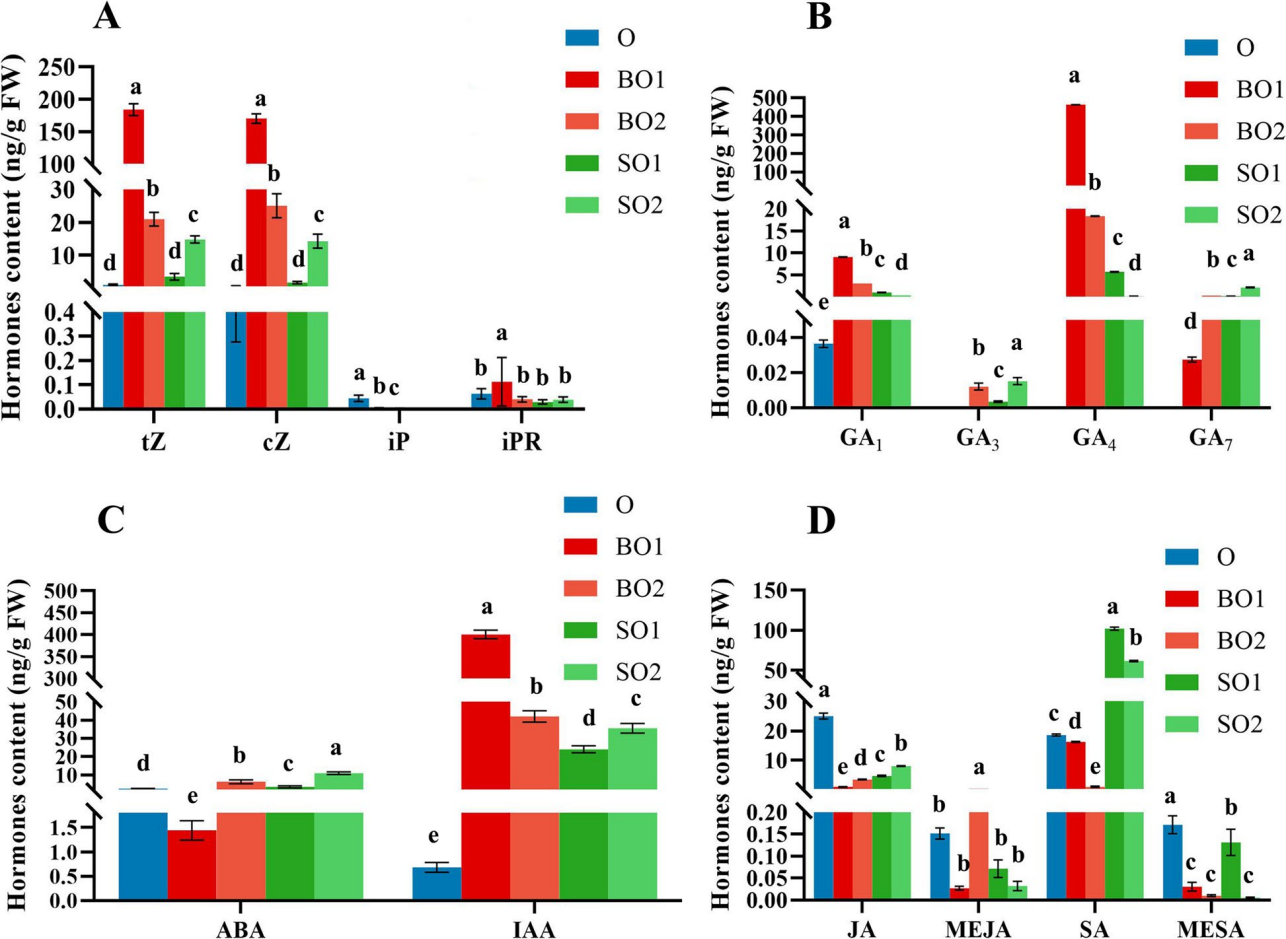
Changes of endogenous hormones in fertile ovules and abortive ovules of *C. henryi*. (**A**) Changes of cytokinins in fertile ovules and abortive ovules. (**B**) Changes of gibberellins in fertile and abortive ovules. (**C**) Changes of abscisic acid and auxin in fertile and abortive ovules. (**D**) Changes of salicylic acid, methyl salicylate, jasmonic acid and methyl jasmonate in fertile and abortive ovules. O: ovule at 35 DAP; BO1: fertile ovule at 49 DAP; SO1: abortive ovule at 49 DAP; BO2: fertile ovule at 63 DAP; SO2: aborted ovule at 63 DAP. The values represent the means ± standard deviations (*n* = 3). Different small letters indicate significant differences of the means (*P* < 0.05). IAA: indoleacetic acid; tZ: trans-Zeatin; cZ: cis-Zeatin; iP: isopentenyladenine; iPR：isopentenyladenosine; GA: gibberellin; ABA: abscisic acid; SA: salicylic acid; MESA: methyl salicylate; JA: jasmonic acid; MEJA: methyl jasmonate

Several key endogenous hormones, including indoleacetic acid (IAA), trans-Zeatin (tZ), cis-Zeatin (cZ), isopentenyladenine (iP), isopentenyladenosine (iPR), GA (1,3,4,7), abscisic acid (ABA), salicylic acid (SA), and jasmonic acid (JA), were measured during seed formation. The contents of CK and IAA in fertile ovules were higher than those in abortive ovules and those in O (ovules at 35 DAP) (Fig. 4). The levels of ABA, SA, and JA in aborted ovules were higher than those in fertile ovules at the same period (Fig. 4). GA_1_ and GA_4_ are biologically active GAs that play major roles in plants [27], and were more abundant in fertile ovules than in abortive ovules. Moreover, the content of GA_4_ was substantially higher than that of GA_1_ (Fig. 4B).

### RNA-seq of the *C. henryi* ovule transcriptome

Fifteen cDNA libraries were generated and sequenced spanning the selected three stages (35 DAP, 49 DAP, 63 DAP) of ovule formation, three repetitions for each stage. After eliminating the adaptor sequences, ambiguous reads, and low-quality reads, with a total of 967.86 GB reads were mapped to the *C. henryi* genome. And the GC contents ranged from 44. 22–45. 94% and the Q30 values were all greater than 90%. Supplementary Fig S2 depicts the length distribution of the *C. henryi* reads. Following that, all clean reads were subjected to a de novo assembly, resulting in 254, 102 transcripts. This indicates that the amount and quality of transcriptome sequencing data from *C. henryi* ovules are relatively high, and that these data can be used to provide a raw data basis for subsequent data assembly.

### Unigene assembly, annotation and analysis

Unigene sequences were annotated using BLAST software in the following five databases: NR (NCBI non-redundant protein sequences), GO (gene ontology), KEGG (Kyoto Encyclopedia of Genes and Genomes), Swiss-Prot, and egg NOG (evolutionary genealogy of genes) for comparison. Annotations were made for 59,521 genes. A total of 29,815 genes had been mapped to specific GO term, which were further classified into 50 terms falling under three categories: biological process (23 terms), cellular component (16 terms), and molecular function (11 terms) (Supplementary Fig S3). To discover active biological pathways involved in ovule formation, the collected genes were linked to the KEGG protein database. These genes were classified into 19 terms (Supplementary Fig S4). The top three most enriched KEGG pathways were 'transcription', 'carbohydrate metabolism', and 'folding, sorting, and degradation'.

### Analysis of the expression levels and differentially expressed genes

**Fig. 5:**
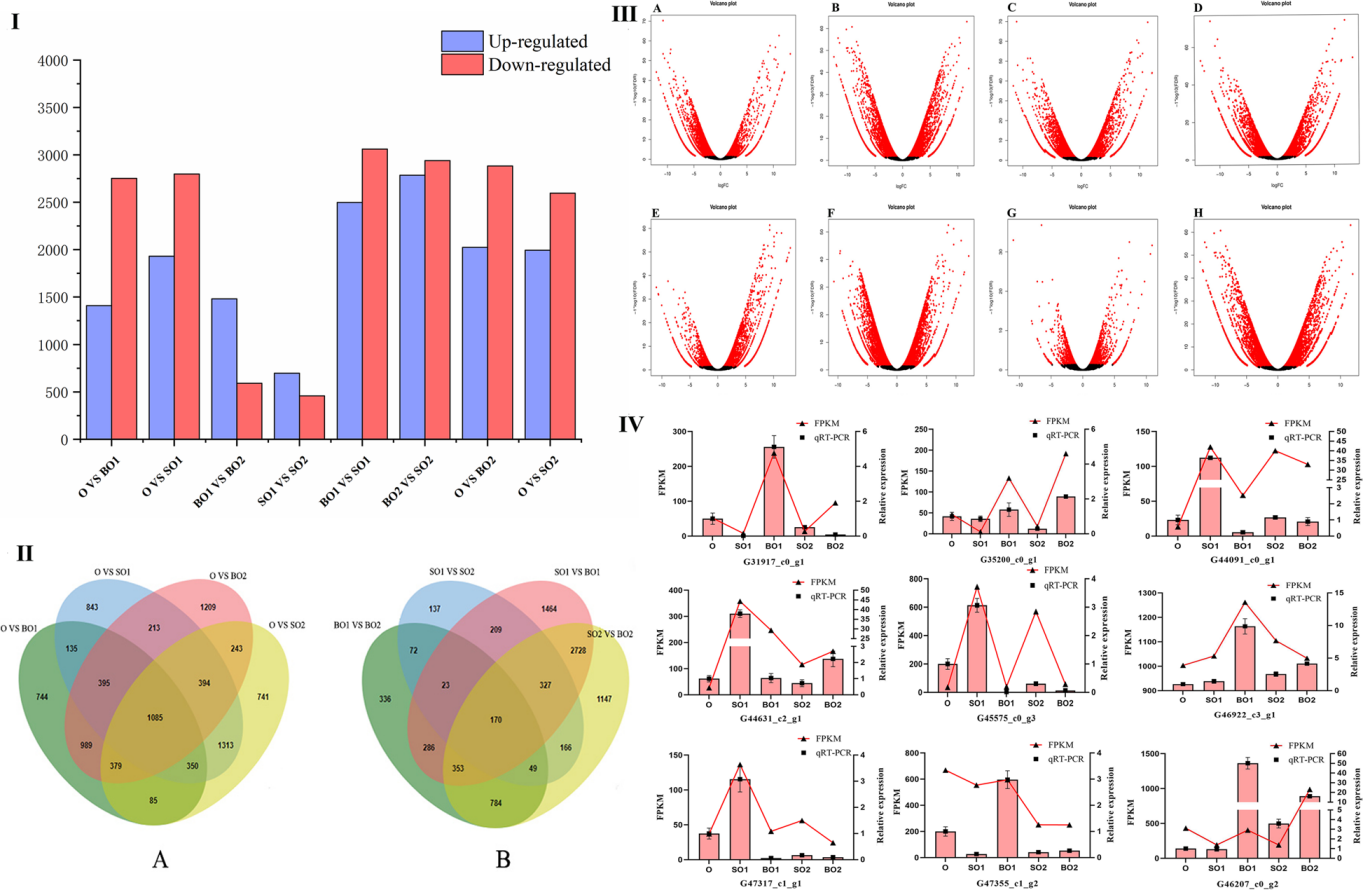
**I** Differentially expressed genes (DEGs) at three selected stages of ovule development. The blue and red columns indicate up-regulated and down-regulated DEGs, respectively. **II** Venn diagrams comparing significantly DEGs that overlap between different selected stages of the ovule development of the *C. henryi*. (A) O VS BO1, O VS BO2, O VS SO1 and O VS SO2. (B) BO1 VS BO2, SO1 VS SO2, SO1 VS BO1, and SO2 VS BO2. **III** Volcano plots of the transcriptome between O VS BO1 (A), O VS BO2 (B), O VS SO1 (C), O VS SO2 (D), BO1 VS BO2 (E), SO1 VS SO2 (F), SO1 VS BO1 (G), and SO2 VS BO2 (H). The statistical significance (log10 of *p*-value; Y-axis) was plotted against log2-fold change (X-axis). The center of the volcano represents a fold change of zero, while the sides indicate down-regulation and up-regulation. **IV** Validation of the expression patterns of differentially expressed genes selected from the RNA-seq analysis by qRT-PCR. The selected gene expression levels derived by the fragments per kilobase per million reads method are shown on the left Y-axis, while the relative gene expression levels determined by qRT-PCR are shown on the right Y-axis. O: ovule at 35 DAP; BO1: fertile ovule at 49 DAP; SO1: abortive ovule at 49 DAP; BO2: fertile ovule at 63 DAP; SO2: aborted ovule at 63 DAP. DAP: days after pollination

Differentially expressed genes (DEGs) were analyzed to identify starch metabolism candidate genes. The DEG-seq program was used to investigate the expression levels of genes involved in *C. henryi* ovule development. After filtering, a transcriptome analysis was performed on 4162, 4728, 4907, 4590, 2073, 1153, 5560, and 5724 genes, and comparisons were made between the ovule (O) VS the fertile ovule (BO1), O VS the abortive ovule (SO1), O VS the fertile ovule (BO2), O VS the aborted ovule (SO2), BO1 VS BO2, SO1 VS BO1, SO1 VS SO2, and SO2 VS BO2. The SO1 VS SO2 comparison produced the fewest DEGs (696 genes up-regulated and 456 genes down-regulated), while the SO2 VS BO2 comparison produced the most DEGs (2785 genes up-regulated and 2939 genes down-regulated) (Fig. 5I). A total of 1058 DEGs were common in all ovule developmental comparisons. Only 170 DEGs were identical during the development of fertile and abortive ovules (Fig. 5II). The significant DEGs are depicted by red dots on volcano plots, which were created to identify the transcripts that were significantly changed throughout ovule development (Fig. 5III).

### Validation of the selected DEGs by qRT-PCR

Quantitative real-time PCR (qRT-PCR) was used to validate some of the DEGs and ensure that the RNA-seq data was reliable. Nine genes were chosen at random and subjected to qRT-PCR analysis. The fact that the qRT-PCR data were mostly consistent with the high-throughput results, implies that the high-throughput sequencing data were trustworthy (Fig. 5IV).

### Identification of genes critical for starch metabolism in *C. henryi* ovules

Many key genes involved in the metabolism of starch and sucrose have been identified. AGPase, GBSS, SBE, and nucleotide pyrophosphatase (NPPS) are some of the enzymes involved in plant starch metabolism. During ovule development, genes encoding key enzymes took part in starch metabolism displayed distinct expression patterns. According to the known starch and sucrose metabolism pathways [12, 28], ten classes of 37 functional genes were identified in our transcriptome database (Supplementary Table S3).

**Fig. 6:**
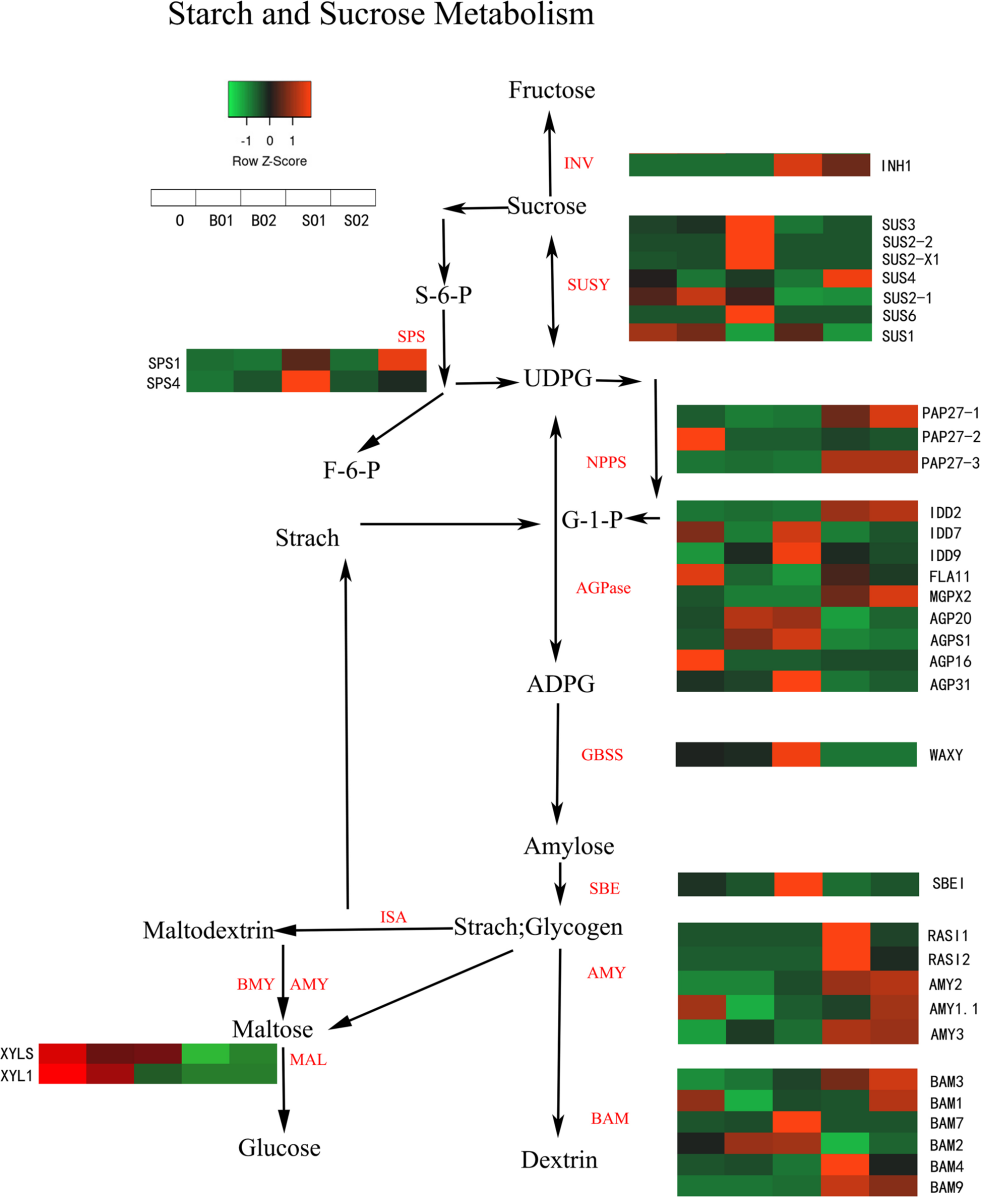
Genes that might be involved in starch and sucrose metabolism and their expression level. The value of log2 (FPKM + 1) is represented using the depth of color, with red representing the up-regulated genes and green representing the down-regulated expression genes. FPKM means the fragments per kilobase of exon per million fragments mapped. INV–invertase; SuSy–sucrose synthase; SPS-sucrose phosphate synthase; NPPS–nucleotide pyrophosphatase; AGPase–ADP-glucose pyrophosphorylase; GBSS–granule-bound starch synthase; SBE–starch-branching; AMY–alpha-amylase; BAM–beta-amylase; ISA–isoamylase; MAL–alpha-glucosidase. O: ovule at 35 DAP; BO1: fertile ovule at 49 DAP; SO1: abortive ovule at 49 DAP; BO2: fertile ovule at 63 DAP; SO2: aborted ovule at 63 DAP. DAP: days after pollination

In the initial step of starch synthesis, AGPase catalyzes the conversion of glucose-1-phosphate into ADP-glucose by consuming ATP and participates in the synthesis of amylose and amylopectin [29, 30]. Through bioinformatic analyses, we identified nine AGPase homologs in the annotated *C. henryi* transcriptome gene dataset. Among these AGPases homologs, we found that three genes were highly expressed in fertile ovules (BO1 and BO2), with much higher levels than those in aborted ovules (*AGPS1*, *AGP31*, and *AGP20*). The FPKM values were > 900 for *AGP31* in BO2 and > 100 for *AGPS1* and *AGP20* in fertile ovules. The expression levels of *MGPX2* and *IDD2* in abortive ovules were higher than those in fertile ovules at the same time (Fig. 6).

GBSS is a key enzyme in amylose synthesis. It can add glucose residues in the adenosine diphosphate glucose to the non-reducing end of glucan through the α-1, 4-D-glycosidic bond, and extend the linear chain of glucan [31]. In our results, a GBSS gene (*WAXY*) was differentially expressed that the expression levels of BO2 were significantly higher than those of SO2. Nucleotide phosphodiesterases (NPPS) can catalyze the hydrolytic breakdown of pyrophosphate and phosphodiester bindings of many nucleotides and nucleotide sugars [32]. In our dataset, three NPPS genes were shown, one of which was tremendously expressed in SO1 and SO2 (*PAP27-1*). The expression of *PAP27-2* was the highest in O. In addition, the expression of the starch branching enzyme (SBE) gene (SBEI) in BO2 was higher than that in other ovules. SBE can cleave the α-1,4-glycosidic bond of the α-1,4-glucan linear donor and simultaneously catalyze the α-1,6-glycosidic bond between the cleaved short chain and the acceptor chain form, resulting in branches [33].

In this research, five AMY genes, six beta-amylase genes and two genes encoding MAL were identified in our predicted database. Two out of the five genes encoding AMY were highly expressed in SO1 (*AMYX3* and *RASI1*). Except for *AMYX3*, whose expression levels in SO1 and SO2 were higher than 100, the remaining AMY genes were expressed at low levels. The expression levels of these two MAL genes in the O samples were higher than 100, which was significantly higher than that in the other samples (Fig. 6). Almost all beta- amylase genes showed higher expression levels in abortive ovules than in fertile ovules. The expression levels of β- amylase-related genes in aborted ovules were higher than those in fertile ovules, and the expression levels of *BAM9* and *BAM4* in SO1 were significantly higher than those in other ovules. The *BAM9* expression in SO2 was 18 times higher than that in fertile ovules (BO2) at the same time. These results indicated that beta-amylase might be a vital starch-degrading enzyme during the formation of fertile and abortive ovules.

Sucrose synthase (SuSy) mainly functions to decompose sucrose into UDP-glucose and fructose, providing precursors and substrates for polysaccharides, cell walls, and starch synthesis. In addition, SuSy can catalyze the reversible conversion of UDP-glucose and fructose to sucrose [27, 34]. Sucrose is hydrolyzed into glucose and fructose by invertase (INV). Sucrose phosphate synthase (SPS) converts UDP-glucose and fructose-6-phosphate to sucrose-6-phosphate [35]. We identified seven SuSy genes in the transcriptome gene dataset. The expression level of *SUS1* was similar in fertile ovules and abortive ovules at all time, and showed a gradual downward trend. Only one of these genes was highly expressed during the BO2 stage (*SuS2-2*). The INV gene (*INH1*) was also highly expressed in abortive ovules, especially in SO1. In this study, we also annotated two SPS genes. However, all SPS genes expression levels were very low in all ovule samples (Fig. 6).

### Changes in the activities of key starch enzymes during the formation of single seeds

**Fig. 7:**
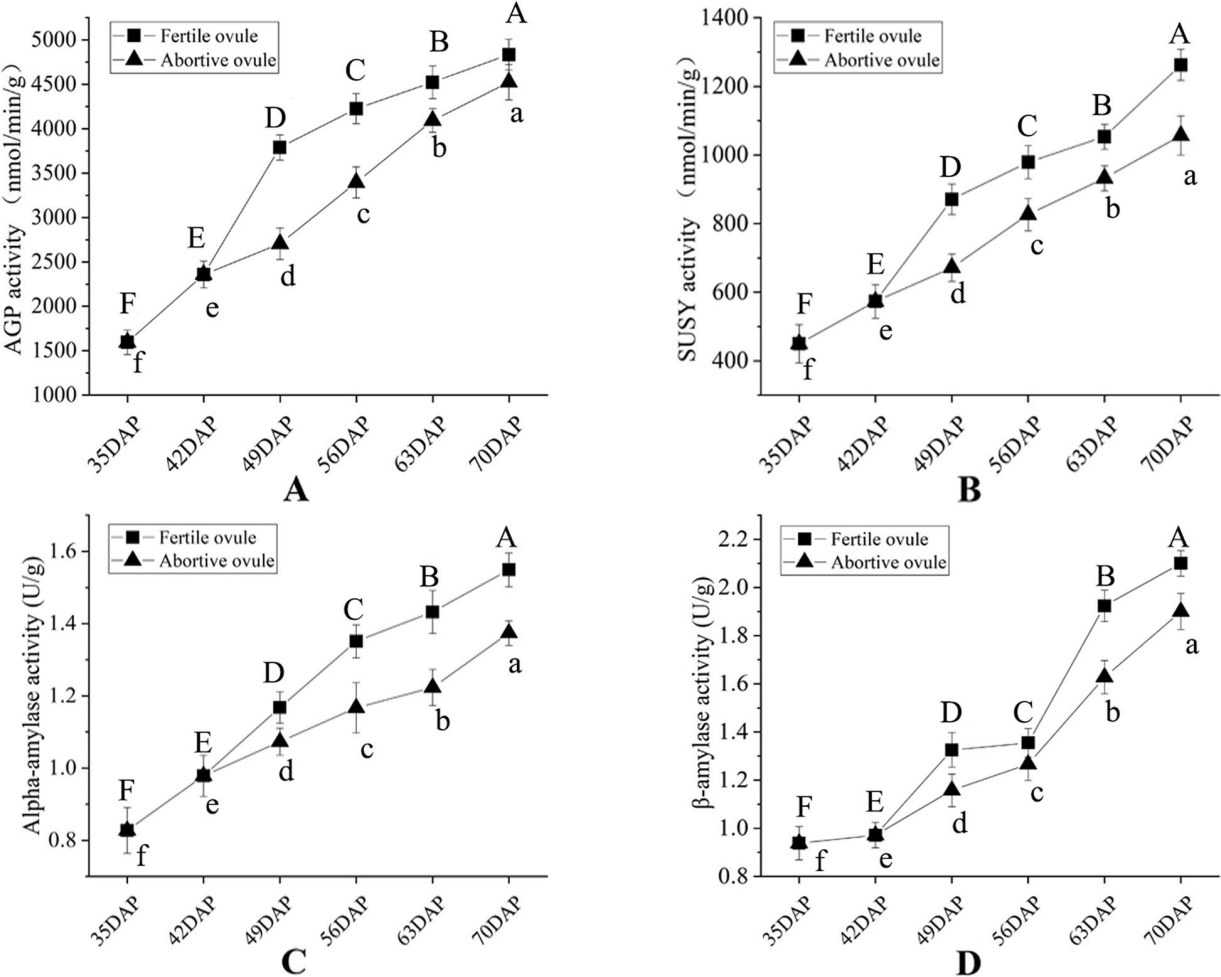
Changes of key enzyme activities of starch in fertile ovules and abortive ovules of *C. henryi*. (**A**) The activity changes of ADP-glucose pyrophosphorylase (AGP) in fertile and abortive ovules. (**B**) The activity changes of sucrose synthase (SUSY) in fertile ovules and abortive ovules. (**C**) The activity changes of alpha-amylase (α- amylase) in fertile ovules and abortive ovules. (**D**) The activity changes of beta-amylase (β-amylase) in fertile ovules and abortive ovules. DAP (days after pollination). The values represent the means ± standard deviations (*n* = 3). Different lowercase letters indicate significant differences between the means (*P* < 0.05). The figure shows the analysis of the significant differences within the group. Significant differences in fertile ovules are marked with capital letters above the dotted line, and differences in aborted ovules are marked with lowercase letters below the line

Considering the results of the differential gene analysis, we selected four representative key enzymes (AGPase, SUSY, α-amylase, and beta-amylase) with high activities for content determination. At 35–70 DAP, the changes of AGPase, SUSY, and AMY were roughly the same and all showed an overall upward trend. Enzyme activities in fertile ovules were higher than those in abortive ovules (Fig. 7A-C). The change in beta-amylase activity was different from that of the above three enzymes. Beta-amylase activity in aborted ovules showed an overall upward trend and increased rapidly after 56 DAP. In contrast, the rate of beta-amylase activity in fertile ovules at 49–56 DAP abruptly decelerated with little change. The beta-amylase activities of aborted and fertile ovules were very similar at 56 DAP (Fig. 7D). We found that the changes in beta-amylase activity were consistent with the changes in starch distribution in the fertile ovules. This further suggests that beta-amylase plays an important role in starch metabolism during the development of the ovule of *C. henryi*.

### Identification of genes critical for hormone biosynthesis and signal transduction in *C. henryi* ovules

**Fig. 8:**
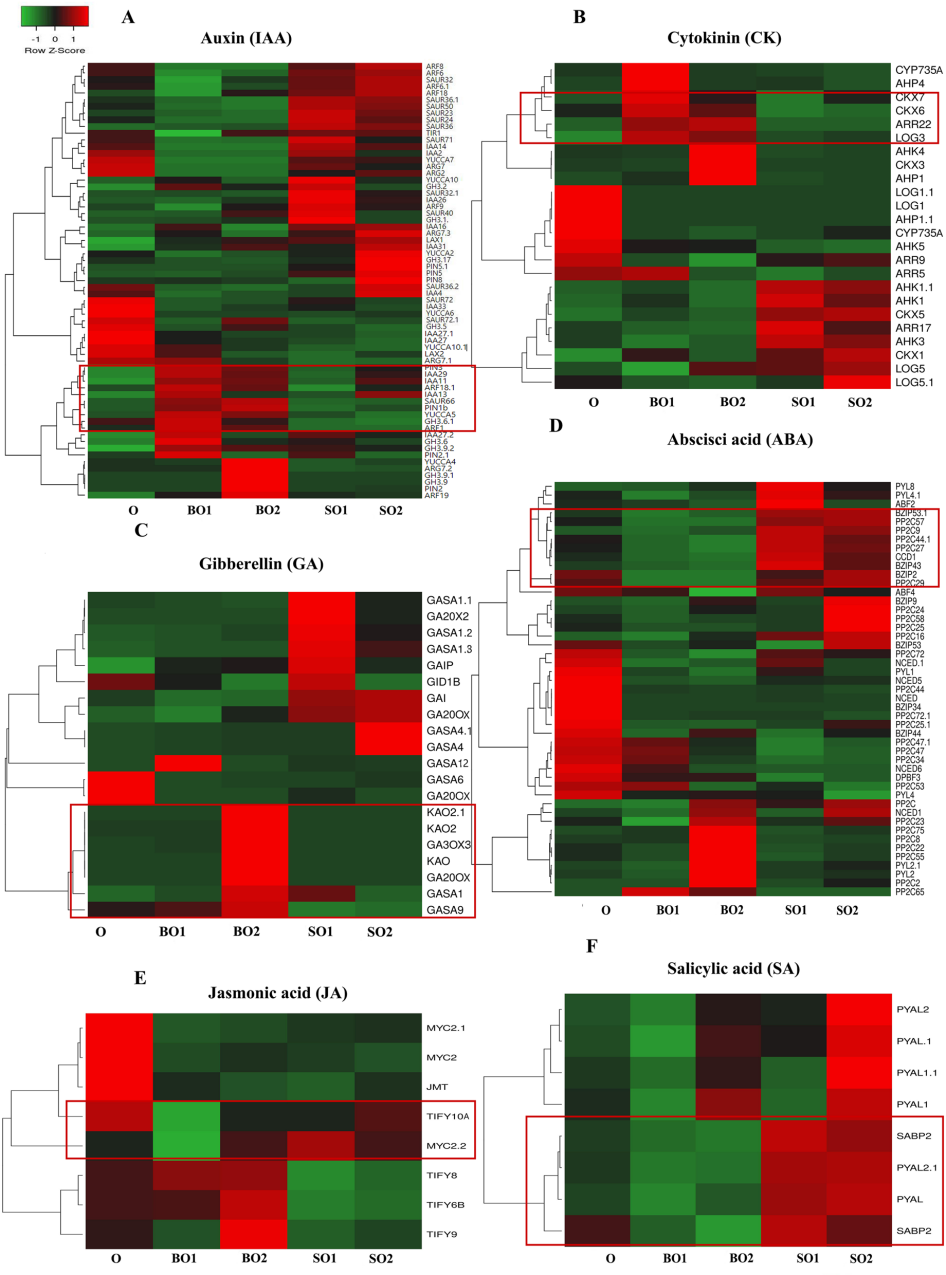
Expression patterns of DEGs related to hormone biosynthesis during single seed formation (**A**–**F**). The changing trend of the expression of DEG circumscribed in red was consistent with the changing trend of the content of the corresponding hormone in the ovule. O: ovule at 35 DAP; BO1: fertile ovule at 49 DAP; SO1: abortive ovule at 49 DAP; BO2: fertile ovule at 63 DAP; SO2: abortive ovule at 63 DAP. DAP: days after pollination

Ovule development is regulated by a complex network of hormones. To better understand hormonal regulation during single seed formation in *C. henryi*, we focused on the DEGs associated with IAA, CK, GA, ABA, JA, and SA synthesis and signaling. We identified 81 DEGs involved in hormone biosynthesis and signal transduction (Supplementary Table 5). Expression trends for a number of DEGs in red border were consistent trends with corresponding trends in hormone contents in ovules (Fig. 8). Most of these differentially expressed genes were associated with IAA and ABA. Four auxin response factor (*ARF*) genes (*ARF8, 6, 6.1, 18*), one *IAA* gene (*IAA14*), and six *SUAR* genes (*SUAR36, 36.1, 23, 24, 32, 50*) were found to be upregulated in abortive ovules (SO1 and SO2). Among these, *ARF6* was highly expressed in abortive ovules (SO1 and SO2), with three times higher expression than that of fertile ovules at the same time. Two IAA genes (*IAA11, 29*), *PIN(3,1b)*, *ARF18.1*, and *YUCCA5* were upregulated during the formation of fertile ovules (BO1 and BO2) (Fig. 8A).

The main physiological functions of CKs are the promotion of cell division and regulation of cell differentiation, delaying aging, and the degradation of proteins and chlorophyll. *CKX (5*, *1)*, *AHK (1, 3, 1.1)*, and *ARR17* were upregulated in SO1 and SO2. *CKX5* was highly transcribed in SO1 and SO2 (FPKM value of > 1300). Similarly, *ARR22*, *CKX6*, and *LOG3* were upregulated during the formation of fertile ovules (BO1 and BO2); however, the expression levels were not high.

With regard to GAs, *GA20X2*, three *GASA (1.1, 1.2, 1.3)*, *GID1B*, and *GAIP* were strongly activated in the GA signaling pathway in SO1. *GAI* and *GA20OX* were consistently upregulated in aborted ovules. The expression level of *GASA4* in SO2 was as high as 1500. The *GASA* family had the highest expression in ovules compared to other families (Fig. 8). A total of 47 DEGs associated with the ABA signaling pathway were identified. Four *PP2C* genes, two *BZIP* genes and *CCD1* were highly expressed in abortive ovules. The expression levels of differential genes related to ABA biosynthesis in aborted ovules were higher than that those in fertile ovules. These results suggest that ABA may plays a negative regulatory role in the formation of single seeds (Fig. 8).

With regard to JA, *TIY8* and *MYC2.2* were upregulated in fertile and abortive ovules, respectively. All DEGs related to SA biosynthesis were up-regulated in aborted ovules (SO1 and SO2) (Fig. 8F).

### Screening of key genes in the hormone biosynthesis pathway

To explain the relationship and interaction between the changes in endogenous hormones and the expression of genes related to hormone synthesis and signal transduction during the formation of single seeds of *C. henryi*, we analyzed the correlation between hormone concentration and its metabolism-related genes. The results showed that the concentrations of *GA20OX1*, *KAO*, *KAO2*, *KAO2.1*, *GA3OX3*, and GA were significantly positively correlated. *PP2C24* and *BZIP9* were significantly positively correlated with ABA concentration while *AHP4* and *CYP735A2* were positively correlated with CK. *TIR16* was significantly negatively correlated with IAA concentrations (Table 2). *MYC2.1*, *MYC2.2*, and *JMT* were significantly positively correlated with the expression level of JA. *SABP2.1* was significantly positively correlated with SA concentration, and its expression level was substantially higher in abortive ovules than in fertile ovules. The above genes may be the key genes of hormone synthesis during the formation of single seed of *C. henryi*.

**Table 2:** Correlations between the concentrations of hormone and its metabolism-related genes

*Variable*	*GA20OX1*	*KAO*	*KAO2*	*KAO2.1*	*GA3OX3*
**GA**	0.9995**	0.9993**	0.9983**	0.9989**	0.9983**
**Variable**	***NCED1***	***PP2C***	***PP2C24***	***PP2C58***	***BZIP9***
**ABA**	0.9178*	0.8785*	0.9722**	0.8891*	0.9923**
**Variable**	***CKX7***	***AHP4***	***CYP735A2***		
**CK**	0.9380*	0.9817**	0.9839**		
**Variable**	***GH3.6***	***TIR1***			
**IAA**	0.9130*	-0.9821*			
**Variable**	***MYC2.1***	***MYC2.2***	***JMT***		
**JA**	0.9554*	0.9952**	0.9385*		
**Variable**	***SABP2.1***				
**SA**	0.9041*				

^*^ *p* < 0.05, ** *p* < 0.01

## Discussion

The cone chestnut fruit has important economic value and is rich in nutrients. Ovule development plays an important role in fruit production. Previous research has mostly focused on the anatomical study of ovule development and the mechanism of ovule abortion. However, little is known about molecular mechanism during single seed formation. In particular, the underlying mechanisms in the metabolism of starch and hormone regulation during *C. henryi* ovule development remain unknown, and the expression levels of the genes involved in starch metabolism and hormone regulation have not been published.

### Starch metabolism during single-seed formation

As one of the major carbohydrates, starch is actively involved in metabolic processes in embryos. Ovule development is accompanied by the accumulation or disappearance of starch and these changes often result in a new developmental stage [36–38]. With regard to the ovule starch metabolism, some plants, such as sweet cherries [39] and hazelnuts [40], have less starch accumulation in aborted ovules, possibly attributed to insufficient starch content that leads to ovary abortion. In the present study, the starch distribution in fertile ovules showed a sudden decrease at 49–56 DAP, while there was no starch distribution in the abortion ovules from 56 DAP. This contrasts with the regularity of carbohydrate changes in many plant ovules, such as those in rice, where the amount of starch in the grain increases along with the increasing sugar content from the early stages of growth [41]. It is speculated that this contradiction may be attributed to embryonic differentiation and development, accelerated cell division, starch utilization exceeding accumulation, and starch amount gradually decreasing or even disappearing [42]. It is also possible to convert starch into a form that is more stable for the development of stored ovules. A possible hypothesis supports the notion that in the critical stage of the formation of fertile ovules and abortive ovules, a large amount of storage material needs to be consumed, and starch is first used as the main nutrient for ovule growth and development [43]. Additionally, starch may be connected with the double fertilization and subsequent zygotic development of fertile ovules, which consume many nutrients, including starch [44].

The expression levels of essential enzymes involved in starch and sucrose metabolism were substantially linked with the changes in starch distribution in the *C. henryi* ovules. Ovule development is a complicated process that needs the coordination and cooperation of many critical factors, especially enzymes. All enzymes in the starch synthesis pathway play crucial roles. Three AGPase, three NPPS genes, one SBE gene, and one GBSS gene were highly expressed during the starch synthesis. The AGPase family showed the highest expression among the five samples. We believe that AGPase may be a key enzyme in starch synthesis. ADPG pyrophosphate acidulase isa key rate-limiting enzyme in the starch anabolic pathway and plays an influential role in the starch synthesis process of maize and various plants [45].

Starch can be broken down by various enzymes. In this study, BAM was an important starch-degrading enzyme in the single-seed setting process of *C. henryi*. Its expression level in abortive ovules is much higher than that of other gene families. In other plants, *BAM* has been confirmed to play an important role. Genome-wide analysis identified 9 *BAM* genes in Arabidopsis, 13 in maize, and 10 in rice. *AtBAM4* acts as a chloroplast regulator in Arabidopsis, is potentially responsive to maltose concentration, and can fine-tune the rate of starch degradation [46, 47]. In potato, *StBAM9* interacts with *StBAM1* on the surface of the starch grains for further starch degradation [48, 49]. In tobacco, the *PrtBAM1* gene increases the amylase activity and promotes starch degradation [50].

Previous studies have shown that beta-amylase is the main degrading enzyme responsible for the hydrolysis of stored starch and the degradation of transition starch. Beta-amylase hydrolyzes starch to beta-limiting dextrin and beta-maltose [51, 52]. In the present study, the beta-amylase activity of fertile ovules decreased sharply at 56 DAP. It is speculated that the activity of the 49–56 DAP abortive ovules decreased rapidly due to the rapid decrease in the amount of starch and substrates for beta-amylase hydrolysis [53]. Interestingly, the beta-amylase activity was consistent with the starch distribution in fertile ovules, which gradually increased and decreased with the increasing and decreasing beta-amylase activity, respectively. Therefore, it is considered that critical enzymes, such as beta-amylase, are closely related to starch synthesis in fertile and abortive ovules, which are directly related to starch accumulation. However, the specific role of beta-amylase requires further exploration.

### Hormonal regulation during single-seed formation

**Fig. 9:**
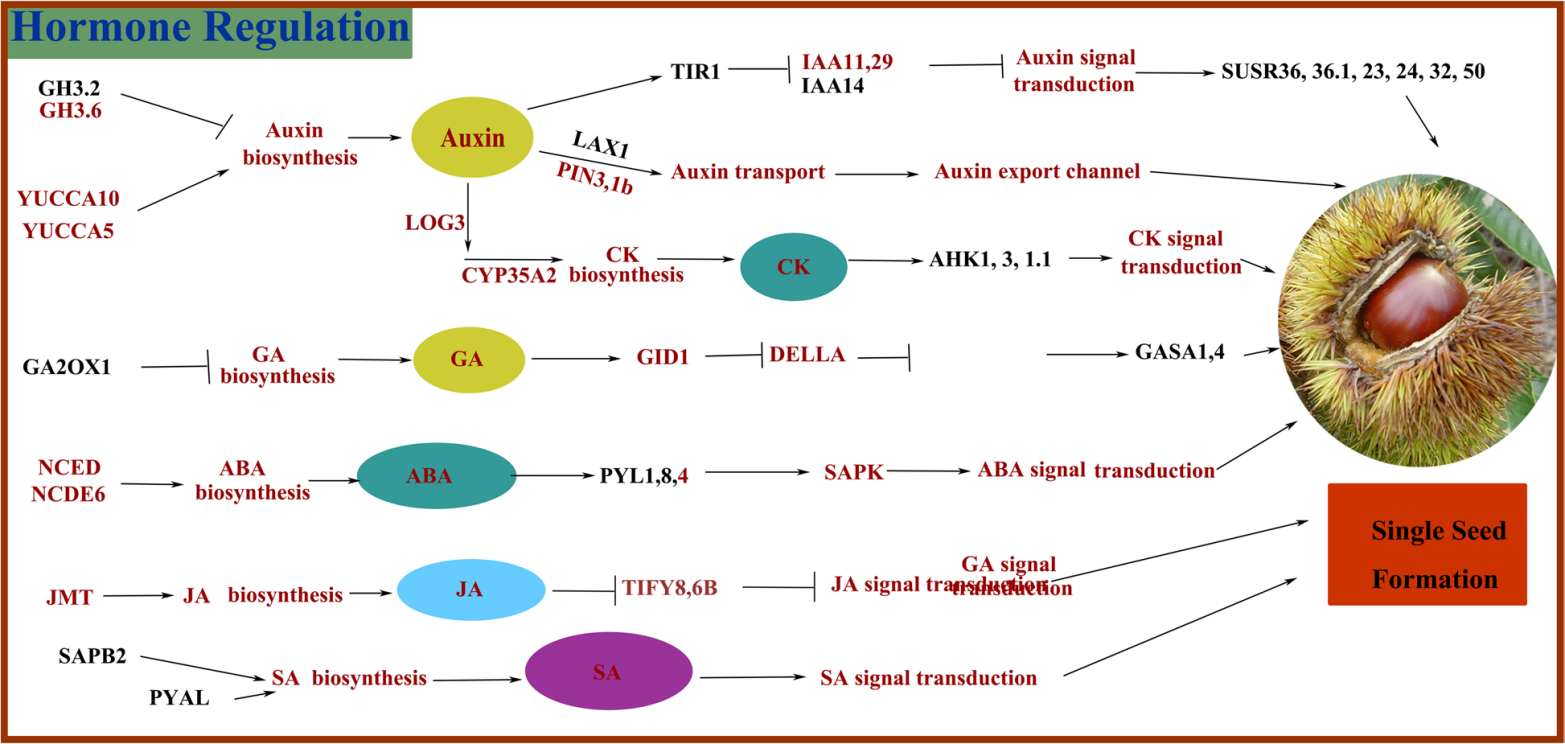
A proposed model to explain endogenous hormone regulation during single seed formation in *C. henryi.* Genes in red font were upregulated in fertile ovules, and genes in black font were upregulated in abortive ovules

Endogenous hormones are an important class of plant growth regulators, and the interaction of a variety of endogenous hormones regulates the ovule development of plants. In this study, we mainly elucidate the regulatory mechanism of GAs, CKs, ABA, and auxin in the formation of single seeds of *C. henryi*. In addition, we further explain the relationship and interaction between single seed formation in *C. henryi* and the key genes involved in hormone biosynthesis and signal transduction (Fig. 9). Multiple studies have shown that GAs play an important role in ovule formation. In a previous study, grapes were sprayed with GA before flowering, and it was found that one of the ways through which GA halted seed development was by damaging the redox system in flowers or berries, which resulted in oxidative damage of seeds and affected the expression of genes related to embryo development [54]. Overexpression of *GASA*4 in *Arabidopsis* has a positive effect on seed size, weight, and yield [55]. In pea, mutations in the GA biosynthesis genes *LH1* and *LH2* greatly affect embryogenesis and lead to the production of sterile seeds [56]. Overexpression of the *GA2OX* gene in pea and *Arabidopsis*, which is responsible for inactivating active of GAs, resulted in seed abortion [57]. In this study, the content of gibberellin in fertile ovules was significantly higher than that in abortive ovules, and the content in fertile embryos of 63 DAP was as high as 467 ng/g. Among them, the content of GA_4_ is the highest, which may play a leading role in gibberellin that promotes the formation of single seed of *C. henryi*. The *GA20X1* was significantly correlated with the gibberellin content during single seed formation in *C. henryi*. The *GASA4* was highly expressed in aborted ovules.

Auxin is mainly involved in regulating cell elongation, differentiation, and division. Auxin has been shown to be involved in regulating the development of the embryo, endosperm, embryo, and seed coat before and after fertilization. The *YUCCA* gene family is a key gene in the process of IAA biosynthesis. In *Arabidopsis*, the auxin biosynthesis enzymes *YUCCA10* and *TARI* are expressed in the endosperm [58]. *PIN1* plays an important role in both ovule initiation and pistil development [59]. A strong loss-of-function allele of *PIN1* induces flower formation without ovules and empty pistils with malformed styles [60]. Previous studies have shown that SAUR is involved in auxin-regulated cell expansion [61]. The expression level of the *YUCCA* gene family was not particularly high in the transcriptome of chestnut ovule. However, *PIN1* was highly expressed in fertile ovules.

Increased pistil CK content can increase pistil morphology and ovule number. CK affects the expression of *PIN1* by promoting the expression of *SPL* and *AG* genes, thereby affecting the development of pistil ovules [62]. In addition to *CKX5*, which is significantly expressed in abortive ovules, other CK genes are expressed in fertile and abortive ovules; however, their expression levels are not high. Interestingly, the content of auxin and cytocatenin reached the highest level at 49 DAP of fertile ovules, which may be in a critical period of growth and development, when cell division and elongation are accelerated.

ABA and GA play antagonistic roles in seed development [63]. Comparing the differential expression of genes at different stages of ovule development between seeded and seedless grapes, it was found that the genes related to ABA synthesis and transduction pathway are upregulated expression in 'seedless' grapes [64]. The content of ABA in aborted ovules was higher than that in fertile ovules, which was consistent with the expression levels of key differentially expressed genes involved in ABA synthesis. Similarly, the content of SA in abortive ovules was substantially higher than that in fertile ovules, and the results were consistent with the expression levels of key differentially expressed genes related to SA biosynthesis. SA may have the same effect as ABA, both of which promote the abortion of ovules and the formation of single seeds.

## Conclusions

In this study, external morphological observations indicated that 49 DAP is a critical period for the formation of fertile and abortive ovules. We investigated the molecular mechanism involved in starch metabolism and the regulation of endogenous hormones in the ovules of *C. henryi* by a transcriptome analysis at three developmental stages. Critical genes related to starch metabolism were further confirmed by enzyme activity assays. We identified ten classes of 37 functional genes related to starch and sucrose metabolic pathways. Among these, beta-amylase may be a crucial starch-degrading enzyme, and the BAM gene may also be an important determinant of the regulation of starch degradation. The contents of auxin, CKs, GAs, and JA in fertile ovules were higher than those in abortive ovules. We also identified and mapped the DEGs associated with hormone synthesis and signal transduction during the formation of single seeds. In summary, we evaluated endogenous hormones and starch metabolism during single seed formation in *C. henryi*, providing a preliminary theoretical basis for explaining the reasons for single seed formation in *C. henryi*.

## Methods

### Plant materials and growth conditions

All methods were carried out in accordance with relevant guidelines. The Chinese chinquapin (*Castanea henryi*) cultivar ‘Huali 4’(XiangS-SC-SH-010–2015) was used in this study, and the pollinated cultivar was ‘Huali 2’(XiangS-SC-SH-008–2015). These plants were planted on the western campus of the Central South University of Forestry and Technology in Changsha, Hunan Province, China. The *C. henryi* fruits were obtained from ten 10-year-old trees at six stages of development (35, 42, 49, 56, 63, and 70 days after pollination, DAP). Twenty seeds were selected for each stage to remove the shell and skin for sampling, fixed in FAA fixative [70% ethanol: glacial acetic acid: formalin (18:1:1, v/v/v)] for 24 h, and then stored in 70% alcohol at 4 °C.

Three representative stages (35 DAP, 49 DAP, 63 DAP) were selected for RNA extraction. The seeds were removed from the fruits, and the shell and skin were removed from the collected seeds. The samples were wrapped in foil, frozen in liquid nitrogen, and kept at -80 °C until they were used for RNA extraction analyses.

### Dynamic growth of fruit and ovule

The developing fruits were measured in the above six stages using a Vernier caliper (Mitutoyo 530, Japan). Twenty fruits were used to measure transverse and longitudinal diameters at each stage. Subsequently, the mean measures were calculated for each period. Using a stereomicroscope (Olympus SZX16, Japan), we photographed and observed the morphology and size of ovules and ovaries.

### Observation of starch distribution

The distribution of starch in the ovules was observed using the paraffin section method combined with periodic acid-Schiff (PAS) staining. Previous research by our group found that the starch grains of *C. henryi* ovules were stained by PAS and their starch grains were purple-red (Supplementary Fig S1 A-C). An inverted microscope (Leica S/M432299, Germany) was used to observe the green fluorescence (460–550 nm). The imaging position of the starch grains was consistent with the position under non-fluorescence imaging, and its color formation effect was better than that of non-fluorescence imaging (Supplementary Fig S1 a–c). Thus, we used an inverted microscope with green fluorescence to photograph and observe ovule starch.

### Measurements of hormone contents

In the present study, ovules with three representative stages (35 DAP, 49 DAP, 63 DAP) were selected for hormone extraction and determination following the methodology established in previous studies [65]. Phytohormones were extracted from approximately 0.5-mg (fresh weight) samples. The GAs, indoleacetic acid (IAA), ABA, c-Zeatin (cZ) and trans-zeatin riboside (tZ), isopentenyladenine (iP), and isopentenyladenosine (iPR) contents of the samples were determined using high performance liquid chromatography (HPLC). Hormone levels was measured using a high performance liquid chromatograph (LC-20AT) with a Hypersil BDS C18 chromatographic column. The flow rate was 1.0 mL·min^−1^.

### RNA extraction and cDNA library construction

Total RNA was extracted from the ovules of the three developmental stages (35 DAP, 49 DAP, 63 DAP) of the *C. henryi*, namely ovule (O) at 35 DAP, fertile ovule (BO1), abortive ovule (SO1) at 49 DAP, fertile ovule (BO2), and aborted ovule (SO2) at 63 DAP. For the cDNA library construction and transcriptome sequencing, the total RNA was extracted using the Plant Total RNA Isolation Kit (Omega, China). We performed extraction with the Microto-Midi Total RNA Purification System according to the manufacturer’s instructions. RNA purity was checked using agarose gel electrophoresis and a NanoDrop ND1000 spectrophotometer (NanoDrop Technologies, Wilmington, DE, USA) [66]. The RNA integrity number (RIN) and rRNA ratio were used to assess RNA concentration and integrity using an Agilent 2100 Bioanalyzer (Santa Clara, CA, USA).

First, qualified samples were taken as total RNA and the fragmentation buffer was poured into mRNA containing oligo (dT) magnetic beads, to fragment the samples. Second, the fragmented mRNA was used as a template. We applied six base random primers to synthesize the first strand of cDNA, and a mixture buffer, dNTPs (A, U, G, C), RNase H, and DNA polymerase I were used to synthesize the second strand of the cDNA chain. Following this step, the cDNA was purified using magnetic beads and eluted using the EB buffer. The fragments were end-repaired and A-tailed [67]. Finally, by using PCR to purify and enrich double-stranded cDNA, a cDNA library was created. Completing the above steps indicated that the preparation of the entire library was complete.

### Unigene assembly, annotation and analysis

On an Illumina NovaSeq 6000 Sequencing System, cDNA sequencing was completed using the paired-end sequencing method. We evaluated the measured raw read data. After the reads’ assembly, we obtained unigene samples of ovules, which were the basis for the gene structure annotation, functional annotation, and expression. Simultaneously, data generated through the RNA-Seq sequencing experiment were compared with the reference genome based on the species' reference genome and gene information. To determine the sequence orientation, we compared and annotated all detected unigene sequences with data samples that contained the NR, GO, KOG number, and KEGG databases [68–70].

### Identification and analysis of differentially expressed genes

The differential expression analysis was performed using the EBSeq software [71]. To analyze the differences in gene expression between different samples, we introduced the FPKM values. The Poisson distribution (parameters: fold change 2 and adjusted *p*-value 0. 001) is used in the DEG seq method. The Q-values were calculated after the *P*-values were corrected. We identified genes with larger than twofold differences and a Q-value ≤ 0.05 and filtered them as significantly differentially expressed genes to increase DEGs accuracy [72, 73]. Because of the limitations of using the database for unigene annotation, a keyword search was performed to identify other differentially expressed genes related to starch metabolism.

### Quantitative real time PCR (qPCR) analysis

To validate the RNA-seq results, nine genes were chosen. The HiScript II Q RT Super Mix for qPCR (+ g DNA wiper) Kit was used to generate the first cDNA after extracting total RNA from five samples subjected to RNA-seq, following the manufacturer’s instructions. Primer 5.0 (Premier, Canada) was used to create gene-specific primers, and the primer sequences are listed in Supplementary Table S4. In a total reaction volume of 20 L, ChamQ Universal SYBR qPCR Master Mix (10 L; Vazyme, Nanjing, China) was mixed with gene-specific primers, sterilized water, and synthesized cDNA. Quantitative PCR was performed using a CFX96 Real-Time System (Bio-Rad, Hercules, CA, USA) according to the manufacturer’s instructions. Because GAPC1 expression is stable throughout ovule development, the internal control gene GAPC1 was used as the reference gene. To acquire relative mRNA expression data, the data were analyzed using the 2^−∆∆Ct^ method. The qRT-PCR analysis was performed with three biological and three technical replicates.

### Determination of starch metabolism-related enzyme activities

Likewise, at six developmental stages (35, 42, 49, 56, 63, and 70 DAP), the ovules were removed from the fruits and immediately stored in liquid nitrogen. We collected various samples of ovules (1 g) at each stage, added the pre-cooled extraction buffer, and homogenized the samples and centrifuged them in an ice bath. The supernatant was collected as the enzyme extract, which was used for the assessment of the α-amylase, beta-amylase, adenosine glucose diphosphate pyrophosphorylase (AGP), and starch synthase (SUSY) activities. The activities of AMY and beta-amylase were homogenized by adding distilled water and extracted according to the method described by Duke et al. [74]. The activities of adenosine diphosphate glucose pyrophosphorylase (AGP) and starch synthase (SUSY) were determined using the Nakamura et al. Method [75]. The activity of each enzyme was measured and thrice.

## Supplementary Information

**Additional file 1: Supplementary Figure S1.** Comparison of ovule starch imaging under non-fluorescent (A-C) and fluorescent photographs (a-c). **Supplementary Figure S2.** Distribution of transcripts lengths.. **Supplementary Figure S3.** GO annotation of genes. **Supplementary Figure S4.** KEGG annotation of genes.

**Additional file 2: Supplementary Table S1.** Statistical analysis of transcriptome data of 5 samples of 3 developmental stages of the *C.* *henryi* ovules. **Supplementary Table S2.** The length distribution of assembled transcripts. **Supplementary Table S3.** Highly and differentially expressed genes involved in starch metabolism in *C.* *henryi* ovules. **Supplementary Table S4.** Primers used in this study. **Supplementary Table S5.** Differentially expressed genes involved in hormonal regulation in *C. henryi* ovules.

## Data Availability

The data of this project were available at NCBI Sequence Read Archive (SRA): PRJNA833559 (https://www.ncbi.nlm.nih.gov/sra/?term=PRJNA833559).
