# Supplementary figures and images for: Comparative analysis of the transcriptome during single-seed formation of Castanea henryi: regulation of starch metabolism and endogenous hormones
Source: BMC Plant Biol. 2023 Feb 13;23:90. doi: 10.1186/s12870-023-04102-4 (PMC9926639; doi:10.1186/s12870-023-04102-4)

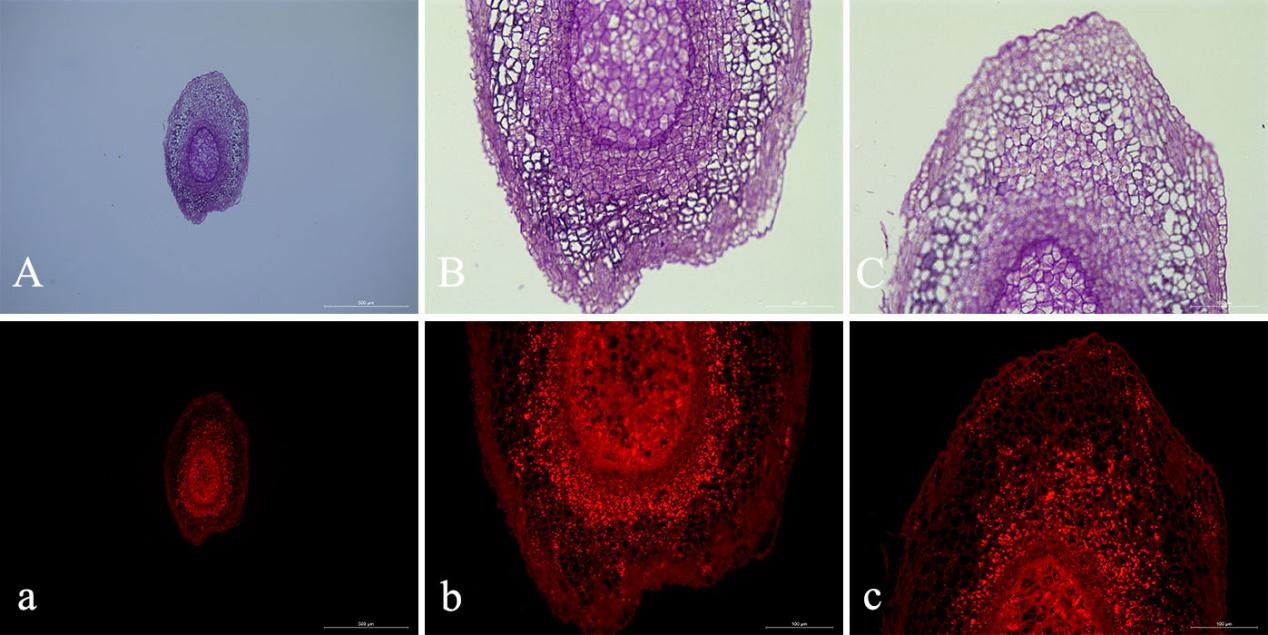

Supplement: Supplementary file 1 — Additional file 1: Supplementary Figure S1. Comparison of ovule starch imaging under non-fluorescent (A-C) and fluorescent photographs (a-c). Supplementary Figure S2. Distribution of transcripts lengths.. Supplementary Figure S3. GO annotation of genes. Supplementary Figure S4. KEGG annotation of genes. [file 12870_2023_4102_MOESM1_ESM.zip › Figure S1.jpg]

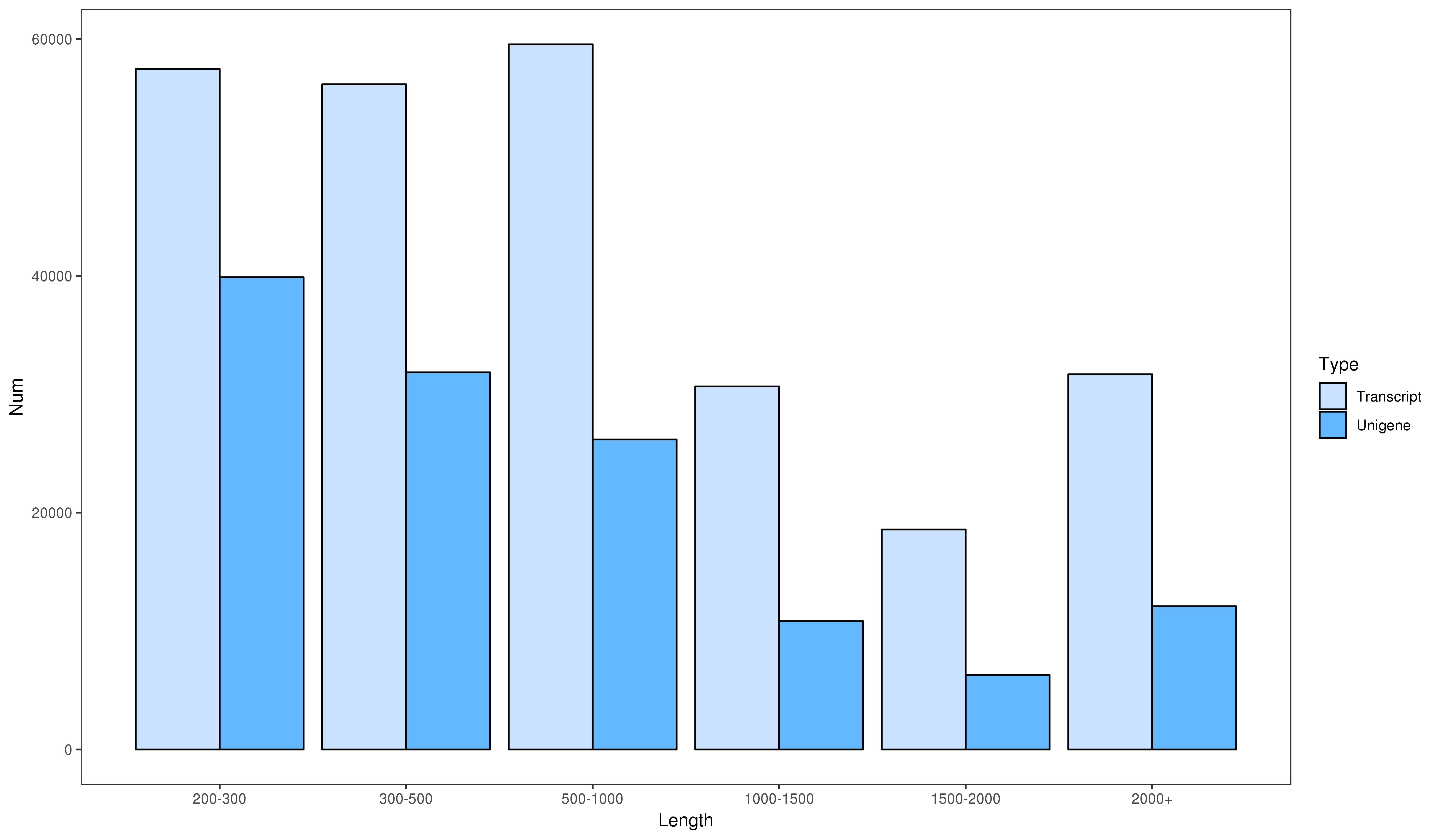

Supplement: Supplementary file 1 — Additional file 1: Supplementary Figure S1. Comparison of ovule starch imaging under non-fluorescent (A-C) and fluorescent photographs (a-c). Supplementary Figure S2. Distribution of transcripts lengths.. Supplementary Figure S3. GO annotation of genes. Supplementary Figure S4. KEGG annotation of genes. [file 12870_2023_4102_MOESM1_ESM.zip › Figure S2.jpg]

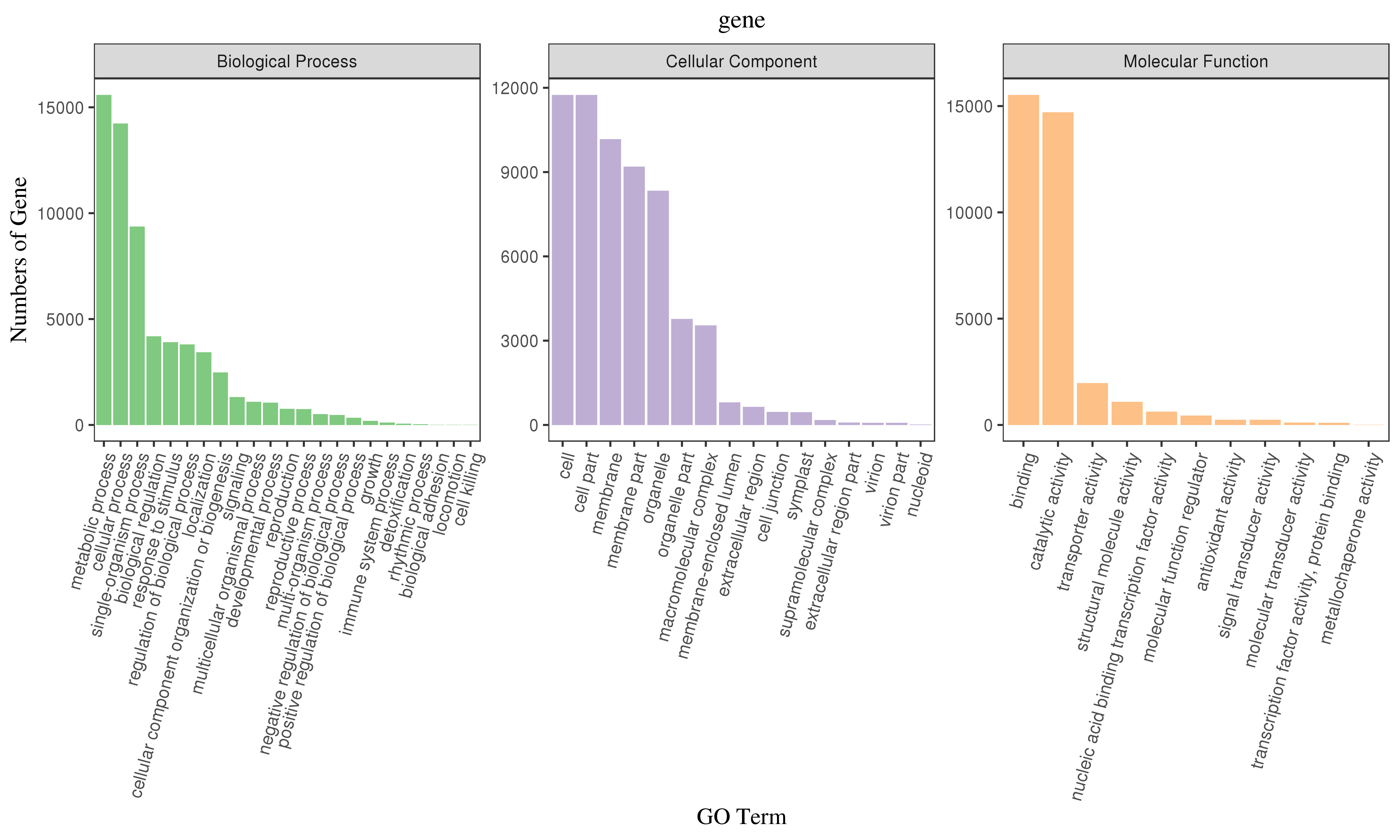

Supplement: Supplementary file 1 — Additional file 1: Supplementary Figure S1. Comparison of ovule starch imaging under non-fluorescent (A-C) and fluorescent photographs (a-c). Supplementary Figure S2. Distribution of transcripts lengths.. Supplementary Figure S3. GO annotation of genes. Supplementary Figure S4. KEGG annotation of genes. [file 12870_2023_4102_MOESM1_ESM.zip › Figure S3.jpg]

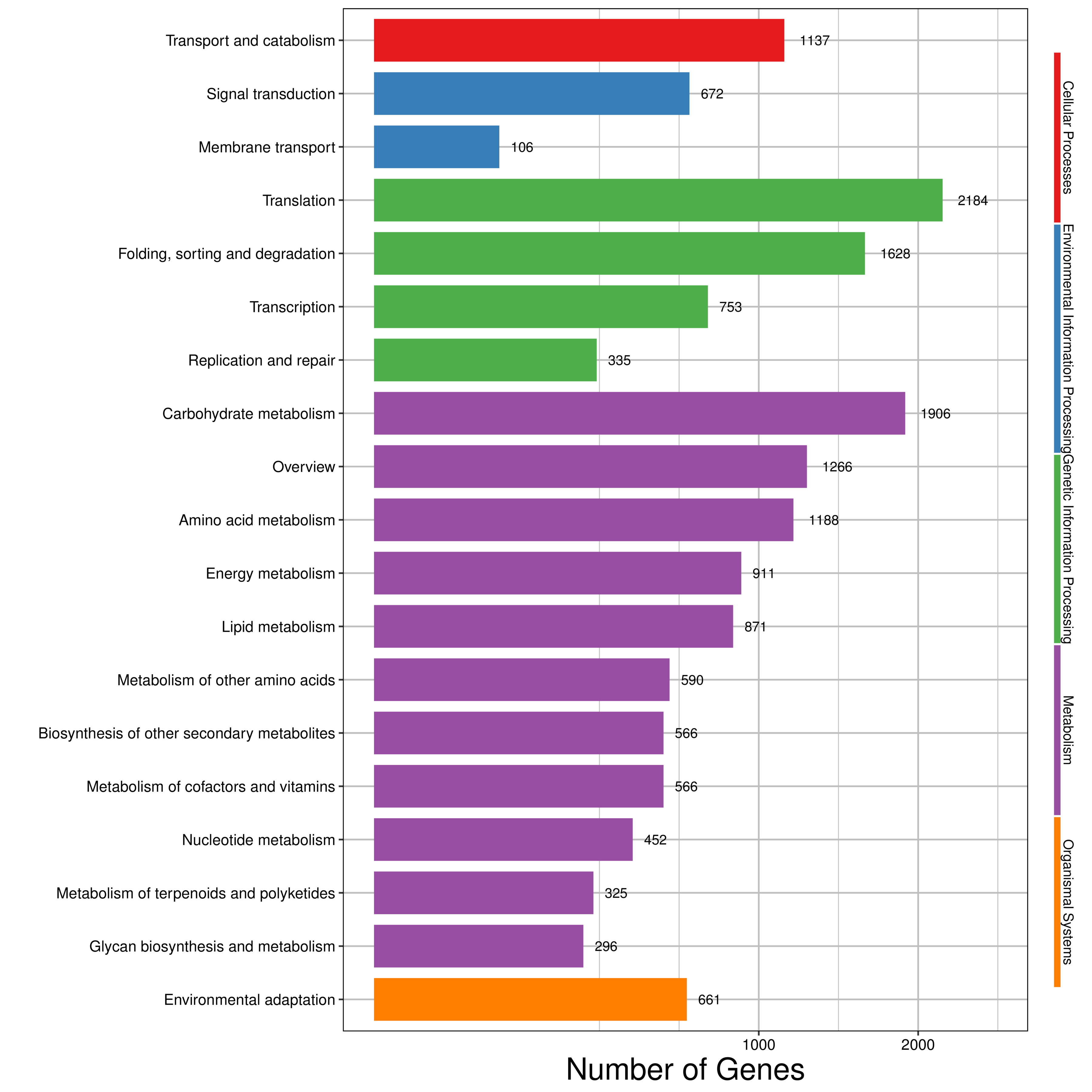

Supplement: Supplementary file 1 — Additional file 1: Supplementary Figure S1. Comparison of ovule starch imaging under non-fluorescent (A-C) and fluorescent photographs (a-c). Supplementary Figure S2. Distribution of transcripts lengths.. Supplementary Figure S3. GO annotation of genes. Supplementary Figure S4. KEGG annotation of genes. [file 12870_2023_4102_MOESM1_ESM.zip › Figure S4.jpg]
