# Supplementary material for: Comparative analysis of the transcriptome during single-seed formation of Castanea henryi: regulation of starch metabolism and endogenous hormones
Source: BMC Plant Biol. 2023 Feb 13;23:90. doi: 10.1186/s12870-023-04102-4 (PMC9926639; doi:10.1186/s12870-023-04102-4)
Supplement: Supplementary file 2 — Additional file 2: Supplementary Table S1. Statistical analysis of transcriptome data of 5 samples of 3 developmental stages of the C. henryi ovules. Supplementary Table S2. The length distribution of assembled transcripts. Supplementary Table S3. Highly and differentially expressed genes involved in starch metabolism in C. henryi ovules. Supplementary Table S4. Primers used in this study. Supplementary Table S5. Differentially expressed genes involved in hormonal regulation in C. henryi ovules. [file 12870_2023_4102_MOESM2_ESM.docx]

**Supplementary Materials:**

**Supplementary Figure S1.** Comparison of ovule starch imaging under non-fluorescent (A-C) and fluorescent photographs (a-c)

**Supplementary Figure. S2.** Distribution of transcripts lengths.

**Supplementary Figure. S3.** GO annotation of genes.

**Supplementary Figure. S4.** KEGG annotation of genes.

**Supplementary Table S1.** Statistical analysis of transcriptome data of 5 samples of 3 developmental stages of the *C. henryi* ovules.

| Sample IB | Clean Reads Sum | Base(M) | Q20% | Q30% | GC% | N% |
| --- | --- | --- | --- | --- | --- | --- |
| SO1_1 | 41,974,098 | 6,226 | 96.06 | 90.2 | 44.6 | 0.00 |
| SO1_2 | 71,349,136 | 10,591 | 96.48 | 90.91 | 44.86 | 0.00 |
| SO1_3 | 50,292,228 | 7,472 | 96.01 | 90.13 | 44.35 | 0.00 |
| SO2_1 | 48,337,702 | 7,175 | 97.17 | 92.46 | 44.7 | 0.00 |
| SO2_2 | 63,083,950 | 9,348 | 97.43 | 92.88 | 44.7 | 0.00 |
| SO2_3 | 63,692,868 | 9,460 | 97.4 | 92.86 | 44.93 | 0.00 |
| BO1_1 | 71,145,574 | 10,583 | 97.71 | 93.35 | 44.82 | 0.00 |
| BO1_2 | 61,967,304 | 9,227 | 97.35 | 92.75 | 44.22 | 0.00 |
| BO1_3 | 73,223,454 | 10,867 | 97.59 | 93.21 | 44.5 | 0.00 |
| BO2_1 | 62,842,382 | 9,339 | 97.66 | 93.14 | 44.86 | 0.00 |
| BO2_2 | 69,358,506 | 10,314 | 97.77 | 93.39 | 44.54 | 0.00 |
| BO2_3 | 72,304,244 | 10,740 | 97.42 | 92.75 | 44.54 | 0.00 |
| O_1 | 74,861,944 | 11,133 | 97.82 | 93.55 | 45.94 | 0.00 |
| O_2 | 77,450,590 | 11,552 | 97.74 | 93.37 | 44.93 | 0.00 |
| O_3 | 65,978,652 | 9,833 | 97.46 | 92.84 | 44.6 | 0.00 |

**Supplementary Table S2.** The length distribution of assembled transcripts.

| **Sequence Size(nt)** | **Transcript Number** | **%** |
| --- | --- | --- |
| 200-300 | 57473 | 22.62% |
| 300-500 | 56177 | 22.11% |
| 500-1000 | 59543 | 23.43% |
| 1000-1500 | 30655 | 12.06% |
| 1500-2000 | 18573 | 7.31% |
| 2000+ | 31681 | 12.47% |
| total | 254102 | 100% |

**Supplementary Table S3.** Highly and differentially expressed genes involved in starch metabolism in *C. henryi* ovules.

| Description | UNIQID | O_FPKM | BO1_FPKM | BO2_FPKM | SO1_FPKM | SO2_FPKM |
| --- | --- | --- | --- | --- | --- | --- |
| AGPase | augustus_masked-scaffold10050-abinit-gene-0.1(AGPS1) | 55.19 | **185.86** | **260.74** | 4.37 | 21.20 |
|  | maker-scaffold05762-augustus-gene-0.12(AGP16) | 481.50 | 19.56 | 19.57 | 49.07 | 38.07 |
|  | maker-scaffold01985-augustus-gene-0.16(AGP31) | 238.63 | **184.83** | **991.20** | 34.01 | 99.73 |
|  | maker-scaffold02231-augustus-gene-0.23(AGP20) | 81.20 | **227.62** | **209.43** | 12.83 | 56.42 |
|  | snap_masked-scaffold12617-abinit-gene-0.3(IDD9) | 3.92 | 14.26 | 32.36 | 13.62 | 11.00 |
|  | maker-scaffold01573-snap-gene-0.44(MGPX2) | 5.22 | 0.92 | 1.51 | 17.32 | 26.18 |
|  | maker-scaffold00235-augustus-gene-0.30(IDD2) | 0.92 | 1.79 | 0.81 | 14.07 | 15.93 |
|  | maker-scaffold03046-snap-gene-0.16(FLA11) | 2.32 | 0.43 | 0.05 | 1.27 | 0.74 |
|  | maker-scaffold06881-snap-gene-0.13(IDD7) | 12.07 | 4.04 | 15.44 | 4.31 | 6.15 |
| GBSS | maker-scaffold03293-augustus-gene-0.20(WAXY) | 432.26 | 404.90 | **978.36** | 191.35 | 191.95 |
| NPPS | augustus_masked-scaffold13577-abinit-gene-0.0(PAP27-1) | 15.62 | 16.00 | 13.98 | **88.96** | **90.95** |
|  | maker-scaffold18614-snap-gene-0.4(PAP27-2) | 0.28 | 0.00 | 0.00 | 0.02 | 0.01 |
|  | augustus_masked-scaffold10193-abinit-gene-0.0(PAP27-3) | 0.10 | 0.04 | 0.06 | 0.32 | 0.50 |
| AMY | maker-scaffold00202-augustus-gene-0.29(AMYX3) | 13.14 | 58.39 | 35.48 | **127.82** | 122.38 |
|  | maker-scaffold01731-snap-gene-0.32(AMY2) | 9.52 | 9.37 | 14.50 | 27.62 | 29.96 |
|  | augustus_masked-scaffold10862-abinit-gene-0.5(RASI1) | 0.00 | 0.08 | 0.00 | **71.93** | 4.76 |
|  | augustus_masked-scaffold24150-abinit-gene-0.0(RASI2) | 0.00 | 0.18 | 0.00 | 15.45 | 3.16 |
|  | maker-scaffold00202-augustus-gene-0.28(AMY1.1) | 3.14 | 0.64 | 1.39 | 1.68 | 3.17 |
| BAM | maker-scaffold07175-augustus-gene-0.11(BAM1) | 65.32 | 19.06 | 36.91 | 36.40 | 71.06 |
|  | maker-scaffold08828-augustus-gene-0.7(BAM9) | 36.44 | 40.34 | 57.37 | **744.04** | 569.80 |
|  | maker-scaffold05952-augustus-gene-0.12(BAM3) | 6.51 | 12.80 | 23.05 | 50.43 | 69.85 |
|  | maker-scaffold00834-snap-gene-0.39(BAM4) | 37.01 | 40.71 | 24.37 | **136.44** | 56.16 |
|  | maker-scaffold01138-snap-gene-0.35(BAM2) | 5.75 | 8.69 | 9.00 | 2.07 | 4.24 |
|  | maker-scaffold05952-augustus-gene-0.12(BAM7) | 0.00 | 0.13 | 2.82 | 0.00 | 0.03 |
| INV | augustus_masked-scaffold12798-abinit-gene-0.0(INH1) | 0.80 | 12.99 | 1.64 | **629.30** | 368.05 |
| SBE | maker-scaffold01810-augustus-gene-0.23(SBEI) | 14.50 | 10.21 | **45.48** | 5.95 | 9.78 |
| SUSY | maker-scaffold10221-snap-gene-0.6(SuS3) | 14.35 | 22.94 | 12.83 | 0.84 | 1.82 |
|  | maker-scaffold00574-augustus-gene-0.33(SuS2-2) | 0.30 | 1.64 | **456.55** | 0.38 | 3.08 |
|  | snap_masked-scaffold06259-abinit-gene-0.9(SuS2-X1) | 0.00 | 0.11 | 5.59 | 0.00 | 0.06 |
|  | MSTRG.24992(SuS4) | 0.06 | 0.05 | 1.75 | 0.00 | 0.00 |
|  | snap_masked-scaffold06259-abinit-gene-0.13(SuS2-1) | 0.77 | 0.91 | 2.97 | 0.33 | 0.66 |
|  | maker-scaffold00005-snap-gene-2.48(SUS6) | 4.75 | 2.52 | 3.97 | 2.59 | 9.09 |
|  | maker-scaffold01256-snap-gene-0.31(SUS1) | 669.23 | **565.75** | 230.00 | **554.02** | 250.89 |
| SPS | maker-scaffold05776-augustus-gene-0.13(SPS4) | 4.03 | 6.13 | 21.47 | 6.12 | 8.58 |
|  | maker-scaffold01885-snap-gene-0.37(SPS1) | 7.68 | 7.33 | 13.99 | 7.66 | 20.83 |
| MAL | maker-scaffold00738-augustus-gene-0.29(XYL1) | 125.19 | 95.93 | 31.14 | 17.07 | 17.72 |
|  | maker-scaffold00738-snap-gene-0.33(XYLS) | 103.46 | 80.53 | 83.48 | 20.52 | 35.00 |

**Supplementary Table S4.** Primers used in this study.

| **Gene ID** | **Gene Name** | **Sequence (5' - 3')** | **Primer**  **Length/bp** |
| --- | --- | --- | --- |
| G46207_c0_g2 | maker-scaffold03293-augustus-gene-0.20 | GCCCATACTATGCCCAGGAACTTG | 24 |
|  |  | CGATACCAGTGATGCCAGTCTTACG | 25 |
| G34572_c0_g2 | maker-scaffold01985-augustus-gene-0.16 | CACTGCACCGAAGACAATCACCTC | 24 |
|  |  | GCACCCTTGAGACCATCGTTGAG | 23 |
| G44091_c0_g1 | maker-scaffold00202-augustus-gene-0.29 | ACCGTGGTGCGTTAGCTGATTG | 22 |
|  |  | GCTGCTTGAAGAATCCCTTTGGTTG | 25 |
| G46431_c1_g5 | augustus_masked-scaffold11995-abinit-gene-0.0 | AGGACACCCTTGGACAGTATGAGAG | 25 |
|  |  | TGCTCCAGGAGACACAATGTTGAAC | 25 |
| G47355_c1_g2 | maker-scaffold01256-snap-gene-0.31 | GCTTCTTAGAATACGGAGGCAAGGG | 25 |
|  |  | ATGTTCTGTTCCACTGACTCGTTCC | 25 |
| G45575_c0_g3 | maker-scaffold08828-augustus-gene-0.7 | GCTGTTGGGAGTTGAAGGTGTAGAG | 25 |
|  |  | GCAAGAGCAAGATAGCCTGACCAC | 24 |
| G46922_c3_g1 | augustus_masked-scaffold00490-abinit-gene-0.3 | GTGGGTGAGAAGTGGCTTGATGAG | 24 |
|  |  | ATGGCGATGAGCGTCAAGTGAAG | 23 |
| G47317_c1_g1 | maker-scaffold00834-snap-gene-0.39 | ATAAGTGTTGGTCTTGGTCCTTCCG | 25 |
|  |  | GCCATCCTCAAGTCCTCCATCATG | 24 |
| G35200_c0_g1 | maker-scaffold01301-augustus-gene-0.25 | GTTCCACCACCACCTGCCAATG | 22 |
|  |  | TTGTTGCTTCTGCTGCTGTTTTGTC | 25 |
| G34941_c0_g1 | maker-scaffold05490-augustus-gene-0.13 | CGGTGAGAGCATCAAGGAAGAACG | 24 |
|  |  | CCCCATTTGCAGGGTCCATAAGC | 23 |
| G44631_c2_g1 | maker-scaffold00851-augustus-gene-0.48 | CGAAGACGATTCCTGGTCACTACG | 24 |
|  |  | GTACTTCCTCACTTGGCGAGTTAGC | 25 |
| G31917_c0_g1 | augustus_masked-scaffold12284-abinit-gene-0.0 | GCTGTTGGGAGTTGAAGGTGTAGAG | 25 |
|  |  | GCAAGAGCAAGATAGCCTGACCAC | 24 |
| reference gene | GAPC1 | CAAGGACTGGAGGGGTGGAAGAG | 23 |
|  |  | ACATCGACAGTTGGAACACGGAAG | 24 |

**Supplementary Table S5.** Differentially expressed genes involved in hormonal regulation in *C. henryi* ovules.

| **Category** | **UNIQID** | **ID** | **O_FPKM** | **BO1_FPKM** | **BO2_FPKM** | **SO1_FPKM** | **SO2_FPKM** |
| --- | --- | --- | --- | --- | --- | --- | --- |
| Auxin | maker-scaffold06891-augustus-gene-0.11 | ARF1 | 51.14 | **100.55** | **73.85** | 26.16 | 26.99 |
|  | maker-scaffold09335-snap-gene-0.11 | ARF6 | 128.24 | 41.89 | 65.55 | **148.08** | **168.06** |
|  | maker-scaffold01422-augustus-gene-0.22 | ARF6.1 | 18.90 | 8.09 | 13.99 | 22.27 | 26.38 |
|  | maker-scaffold03498-augustus-gene-0.29 | ARF8 | 49.71 | 20.02 | 19.62 | **52.44** | **69.18** |
|  | maker-scaffold00232-augustus-gene-0.39 | ARF9 | 34.96 | 14.47 | 47.21 | **85.51** | **48.96** |
|  | maker-scaffold06503-snap-gene-0.17 | ARF18 | 19.00 | 11.65 | 24.66 | **30.07** | **34.27** |
|  | maker-scaffold01755-snap-gene-0.22 | ARF18.1 | 13.95 | 28.58 | 22.96 | 7.90 | 17.11 |
|  | maker-scaffold01829-augustus-gene-0.23 | ARF19 | 14.31 | 25.16 | 41.04 | 23.32 | 21.60 |
|  | maker-scaffold01027-augustus-gene-0.43 | ARG2 | 400.85 | 77.81 | 78.20 | 215.06 | 283.64 |
|  | MSTRG.14895 | ARG7 | 1.05 | 0.18 | 0.17 | 0.76 | 0.51 |
|  | augustus_masked-scaffold03959-abinit-gene-0.4 | ARG7 | 95.50 | 86.71 | 29.53 | 9.80 | 11.87 |
|  | augustus_masked-scaffold09176-abinit-gene-0.2 | ARG7 | 10.87 | 10.38 | 43.74 | 2.86 | 4.79 |
|  | augustus_masked-scaffold11839-abinit-gene-0.0 | ARG7 | 28.48 | 51.59 | 25.29 | 56.73 | 91.10 |
|  | maker-scaffold10316-augustus-gene-0.2 | GH3.1 | 0.04 | 0.19 | 0.70 | 4.08 | 0.34 |
|  | maker-scaffold15213-augustus-gene-0.2 | GH3.5 | 0.35 | 0.03 | 0.16 | 0.05 | 0.00 |
|  | maker-scaffold00288-augustus-gene-0.24 | GH3.6 | 16.47 | **154.57** | **65.42** | 80.32 | 51.44 |
|  | maker-scaffold00803-augustus-gene-0.38 | GH3.6 | 31.78 | 54.87 | 28.62 | 5.43 | 3.37 |
|  | maker-scaffold00838-snap-gene-0.35 | GH3.17 | 0.26 | 0.17 | 0.14 | 0.29 | 0.82 |
|  | maker-scaffold00851-augustus-gene-0.48 | GH3.2 | 26.76 | 246.77 | 166.53 | **357.95** | **115.78** |
|  | maker-scaffold08467-snap-gene-0.9 | GH3.9 | 0.00 | 0.03 | 2.56 | 0.00 | 0.02 |
|  | maker-scaffold08715-snap-gene-0.5 | GH3.9 | 0.01 | 0.00 | 3.84 | 0.00 | 0.03 |
|  | maker-scaffold11822-augustus-gene-0.3 | GH3.9 | 5.71 | **26.18** | **21.89** | 19.92 | 17.48 |
|  | maker-scaffold01620-augustus-gene-0.22 | IAA2 | 10.30 | 0.38 | 0.32 | 10.40 | 3.59 |
|  | snap_masked-scaffold00188-abinit-gene-1.13 | IAA4 | 56.47 | 17.45 | 25.26 | 29.56 | 85.07 |
|  | snap_masked-scaffold03771-abinit-gene-0.12 | IAA11 | 18.23 | **66.69** | **59.33** | 29.46 | 55.46 |
|  | maker-scaffold06130-augustus-gene-0.11 | IAA13 | 4.37 | **78.41** | **31.12** | 22.45 | 69.45 |
|  | maker-scaffold05608-snap-gene-0.19 | IAA14 | 6.90 | 0.45 | 0.46 | **9.73** | **6.96** |
|  | maker-scaffold08184-augustus-gene-0.12 | IAA16 | 117.15 | 228.35 | 101.16 | **261.35** | **285.75** |
|  | snap_masked-scaffold06951-abinit-gene-0.8 | IAA26 | 37.20 | 30.29 | 52.06 | **132.29** | **72.04** |
|  | augustus_masked-scaffold01084-abinit-gene-0.0 | IAA27 | 184.89 | 84.76 | 70.05 | 59.85 | 41.75 |
|  | maker-scaffold09043-snap-gene-0.9 | IAA27 | 289.27 | 128.96 | 90.16 | 91.75 | 65.46 |
|  | maker-scaffold04454-snap-gene-0.11 | IAA27 | 23.04 | 98.56 | 43.37 | 74.68 | 56.29 |
|  | augustus_masked-scaffold00577-abinit-gene-0.15 | IAA29 | 9.01 | **158.35** | **119.20** | 33.28 | 93.47 |
|  | maker-scaffold05540-augustus-gene-0.9 | IAA31 | 2.47 | 6.45 | 8.53 | 6.91 | 11.69 |
|  | maker-scaffold10530-snap-gene-0.6 | IAA33 | 2.11 | 0.00 | 0.26 | 0.56 | 0.14 |
|  | augustus_masked-scaffold00406-abinit-gene-0.3 | LAX1 | 39.67 | 94.48 | 108.23 | **120.16** | **146.80** |
|  | maker-scaffold00132-snap-gene-0.41 | LAX2 | 27.04 | 17.96 | 9.66 | 7.82 | 11.98 |
|  | augustus_masked-scaffold09028-abinit-gene-0.0 | PIN2 | 0.00 | 0.00 | 10.42 | 0.00 | 0.53 |
|  | snap_masked-scaffold09074-abinit-gene-0.6 | PIN2 | 0.15 | 0.68 | 0.03 | 0.36 | 0.03 |
|  | maker-scaffold01555-augustus-gene-0.24 | PIN3 | 7.75 | 51.96 | 41.60 | 15.78 | 30.16 |
|  | augustus_masked-scaffold02805-abinit-gene-0.0 | PIN5 | 0.37 | 0.23 | 0.64 | 2.74 | 6.05 |
|  | maker-scaffold04243-augustus-gene-0.14 | PIN8 | 2.81 | 3.01 | 2.74 | 1.82 | 7.15 |
|  | maker-scaffold02032-augustus-gene-0.11 | PIN1b | 20.32 | **126.23** | **159.12** | 5.79 | 31.13 |
|  | augustus_masked-scaffold00508-abinit-gene-0.1 | SAUR72 | 34.02 | 0.23 | 0.64 | 10.79 | 2.78 |
|  | augustus_masked-scaffold01288-abinit-gene-0.3 | SAUR72 | 2.94 | 0.00 | 1.89 | 0.05 | 0.30 |
|  | augustus_masked-scaffold02206-abinit-gene-0.5 | SAUR71 | 1.20 | 0.06 | 0.13 | 2.22 | 0.90 |
|  | augustus_masked-scaffold03554-abinit-gene-0.8 | SAUR36 | 1.95 | 2.47 | 2.78 | 8.11 | 6.77 |
|  | augustus_masked-scaffold04349-abinit-gene-0.2 | SAUR40 | 0.04 | 0.53 | 6.24 | 13.09 | 1.77 |
|  | augustus_masked-scaffold04443-abinit-gene-0.0 | SAUR23 | 3.15 | 1.27 | 0.00 | **15.54** | **10.76** |
|  | augustus_masked-scaffold05986-abinit-gene-0.4 | SAUR66 | 4.96 | **39.63** | **48.71** | 1.57 | 3.20 |
|  | augustus_masked-scaffold06347-abinit-gene-0.0 | SAUR50 | 3.35 | 0.60 | 0.36 | 7.60 | 6.45 |
|  | augustus_masked-scaffold06962-abinit-gene-0.0 | SAUR36 | 25.67 | 13.15 | 8.06 | **59.01** | **51.42** |
|  | augustus_masked-scaffold24702-abinit-gene-0.0 | SAUR32 | 7.44 | 2.10 | 6.11 | 9.34 | 11.44 |
|  | maker-scaffold04443-snap-gene-0.20 | SAUR24 | 0.33 | 0.00 | 0.00 | 0.92 | 0.57 |
|  | maker-scaffold06114-augustus-gene-0.16 | SAUR36 | 0.23 | 0.02 | 0.00 | 0.06 | 0.35 |
|  | snap_masked-scaffold01546-abinit-gene-0.12 | SAUR32 | 3.45 | 5.27 | 2.52 | **81.37** | **28.84** |
|  | maker-scaffold00791-snap-gene-0.37 | YUCCA4 | 0.45 | 0.29 | 2.29 | 0.08 | 0.27 |
|  | maker-scaffold01622-augustus-gene-0.29 | YUCCA7 | 1.37 | 0.00 | 0.00 | 0.88 | 0.75 |
|  | maker-scaffold01710-snap-gene-0.18 | YUCCA2 | 0.55 | 0.09 | 0.28 | 0.53 | 1.39 |
|  | augustus_masked-scaffold01805-abinit-gene-0.0 | YUCCA6 | 6.43 | 0.37 | 0.41 | 0.32 | 0.79 |
|  | augustus_masked-scaffold03686-abinit-gene-0.2 | YUCCA5 | 2.03 | 9.71 | 7.68 | 2.36 | 0.55 |
|  | augustus_masked-scaffold20874-abinit-gene-0.1 | YUCCA10 | 0.00 | 0.45 | 0.00 | 1.32 | 0.30 |
|  | maker-scaffold05708-snap-gene-0.14 | YUCCA10.1 | 8.67 | 5.02 | 2.74 | 1.40 | 1.13 |
|  | maker-scaffold11370-augustus-gene-0.10 | TIR1 | 13.47 | 1.98 | 13.69 | 15.46 | 14.66 |
| Cytokinin | maker-scaffold02051-snap-gene-0.14 | LOG5 | 1.56 | 0.44 | 2.69 | 2.83 | 3.37 |
|  | maker-scaffold02338-augustus-gene-0.23 | LOG1 | 34.56 | 0.06 | 0.29 | 0.29 | 0.12 |
|  | maker-scaffold02374-snap-gene-0.28 | LOG5 | 0.75 | 0.11 | 0.00 | 0.38 | 2.39 |
|  | maker-scaffold06632-augustus-gene-0.14 | LOG1 | 45.75 | 0.00 | 0.00 | 0.19 | 0.08 |
|  | maker-scaffold07815-snap-gene-0.9 | LOG3 | 0.63 | 7.36 | 6.05 | 2.34 | 2.76 |
|  | MSTRG.16069 | CKX6 | 3.98 | 7.24 | 5.36 | 1.97 | 2.32 |
|  | maker-scaffold05153-snap-gene-0.12 | CKX5 | 117.33 | 413.00 | 263.65 | **1345.15** | **1481.58** |
|  | maker-scaffold05180-augustus-gene-0.8 | CKX3 | 0.04 | 0.03 | 0.79 | 0.02 | 0.02 |
|  | maker-scaffold07807-snap-gene-0.7 | CKX1 | 3.60 | 24.30 | 11.46 | 28.81 | 38.53 |
|  | maker-scaffold12974-augustus-gene-0.4 | CKX7 | 15.74 | 48.72 | 26.69 | 9.76 | 23.51 |
|  | maker-scaffold00003-snap-gene-1.38 | AHK3 | 20.38 | 11.61 | 17.01 | 46.01 | 38.33 |
|  | maker-scaffold01759-augustus-gene-0.23 | AHK5 | 1.28 | 0.50 | 0.46 | 0.09 | 0.03 |
|  | maker-scaffold02321-augustus-gene-0.25 | AHK4 | 2.38 | 1.73 | 17.75 | 0.72 | 1.24 |
|  | maker-scaffold13896-augustus-gene-0.2 | AHK1 | 0.30 | 0.66 | 0.22 | 1.83 | 1.25 |
|  | maker-scaffold22701-snap-gene-0.4 | AHK1.1 | 0.50 | 0.76 | 0.31 | 2.62 | 2.26 |
|  | augustus_masked-scaffold12540-abinit-gene-0.1 | ARR17 | 11.81 | 4.52 | 1.95 | 60.02 | 31.37 |
|  | maker-scaffold00624-augustus-gene-0.30 | ARR9 | 32.17 | 14.40 | 6.73 | 20.26 | 23.23 |
|  | maker-scaffold01610-augustus-gene-0.42 | ARR5 | 12.38 | 13.45 | 3.79 | 1.40 | 3.85 |
|  | maker-scaffold10425-augustus-gene-0.6 | ARR22 | 0.00 | 2.12 | 2.52 | 0.00 | 0.00 |
|  | MSTRG.21984 | AHP4 | 0.21 | 2.07 | 0.25 | 0.32 | 0.05 |
|  | MSTRG.9068 | AHP1 | 0.75 | 1.43 | 7.46 | 0.66 | 0.82 |
|  | augustus_masked-scaffold00264-abinit-gene-0.6 | AHP1.1 | 8.99 | 0.14 | 0.18 | 0.15 | 0.06 |
|  | maker-scaffold13110-snap-gene-0.12 | CYP735A1 | 0.67 | 0.11 | 0.05 | 0.12 | 0.20 |
|  | maker-scaffold02898-snap-gene-0.30 | CYP735A2 | 0.90 | 4.59 | 0.74 | 0.55 | 0.39 |
| Gibberellin | maker-scaffold01649-augustus-gene-0.29 | GA20X2 | 0.37 | 0.44 | 0.52 | 13.92 | 3.16 |
|  | maker-scaffold06826-snap-gene-0.5 | GA20OX1 | 5.38 | 1.89 | 12.16 | 22.96 | 25.58 |
|  | maker-scaffold08490-augustus-gene-0.14 | GA20OX1 | 0.42 | 3.30 | 19.67 | 0.51 | 10.98 |
|  | augustus_masked-scaffold10291-abinit-gene-0.4 | GA20OX2 | 3.42 | 0.00 | 0.00 | 0.48 | 0.00 |
|  | maker-scaffold00514-snap-gene-0.40 | GID1B | 27.03 | 17.02 | 4.11 | 36.36 | 5.50 |
|  | MSTRG.14478 | GASA9 | 55.91 | 69.71 | 116.88 | 2.25 | 8.03 |
|  | augustus_masked-scaffold00563-abinit-gene-0.5 | GASA1 | 0.57 | 2.73 | 18.97 | 12.22 | 1.07 |
|  | augustus_masked-scaffold00563-abinit-gene-1.1 | GASA1 | 9.49 | 7.56 | 2.01 | 150.08 | 32.49 |
|  | augustus_masked-scaffold00563-abinit-gene-1.2 | GASA1 | 0.60 | 2.02 | 3.30 | 34.53 | 10.95 |
|  | maker-scaffold00563-augustus-gene-0.20 | GASA1 | 6.41 | 45.65 | 37.18 | **544.28** | **257.59** |
|  | maker-scaffold01691-augustus-gene-0.15 | GASA4 | 434.53 | 464.07 | 485.51 | **509.99** | **1567.62** |
|  | maker-scaffold01667-augustus-gene-0.15 | GASA4 | 334.53 | 388.07 | 507.51 | **604.99** | **1572.66** |
|  | snap_masked-scaffold01279-abinit-gene-0.26 | GASA6 | 146.78 | 0.15 | 2.94 | 3.46 | 13.35 |
|  | augustus_masked-scaffold01931-abinit-gene-0.0 | GASA12 | 0.23 | 35.27 | 0.00 | 3.98 | 2.17 |
|  | maker-scaffold02448-snap-gene-0.26 | KAO | 2.54 | 3.26 | 581.40 | 2.00 | 3.02 |
|  | augustus_masked-scaffold05498-abinit-gene-0.3 | KAO2 | 0.59 | 0.81 | 24.49 | 0.09 | 0.08 |
|  | snap_masked-scaffold16978-abinit-gene-0.3 | KAO2.1 | 0.39 | 0.38 | 23.62 | 0.00 | 0.10 |
|  | maker-scaffold00146-augustus-gene-1.16 | GA3OX3 | 0.13 | 0.22 | 5.06 | 0.12 | 0.04 |
|  | augustus_masked-scaffold03933-abinit-gene-0.5 | GAI | 56.67 | 37.01 | 38.93 | 103.18 | 112.25 |
|  | maker-scaffold01006-snap-gene-0.25 | GAIP | 29.99 | 75.18 | 80.79 | 134.31 | 71.52 |
| Abscisic acid | MSTRG.19653 | NCED | 15.32 | 0.00 | 0.39 | 0.75 | 0.13 |
|  | augustus_masked-scaffold02175-abinit-gene-0.2 | NCED1 | 4.62 | 1.67 | 14.54 | 2.90 | 16.28 |
|  | augustus_masked-scaffold17811-abinit-gene-0.0 | NCED5 | 22.96 | 3.17 | 2.29 | 7.12 | 6.61 |
|  | maker-scaffold06458-snap-gene-0.7 | CCD1 | 50.81 | 34.56 | 30.44 | 91.00 | 68.88 |
|  | maker-scaffold23959-snap-gene-0.5 | NCED | 2.70 | 0.48 | 0.73 | 1.95 | 0.96 |
|  | snap_masked-scaffold02354-abinit-gene-0.28 | NCED6 | 0.36 | 0.16 | 0.01 | 0.00 | 0.00 |
|  | augustus_masked-scaffold01371-abinit-gene-0.8 | PYL4 | **172.90** | 102.25 | 111.57 | 105.47 | 45.43 |
|  | augustus_masked-scaffold01694-abinit-gene-0.11 | PYL4 | 1.60 | 0.19 | 1.02 | 4.00 | 1.98 |
|  | augustus_masked-scaffold06547-abinit-gene-0.6 | PYL2 | 0.12 | 0.00 | 1.09 | 0.12 | 0.18 |
|  | augustus_masked-scaffold07512-abinit-gene-0.0 | PYL2 | 0.28 | 0.12 | 1.39 | 0.33 | 0.44 |
|  | augustus_masked-scaffold10359-abinit-gene-0.2 | PYL1 | 192.42 | 67.31 | 37.34 | 103.53 | 67.66 |
|  | maker-scaffold01220-augustus-gene-0.21 | PYL8 | 20.63 | 22.62 | 30.51 | 69.44 | 37.43 |
|  | maker-scaffold00806-snap-gene-0.57 | ABF2 | 20.47 | 17.85 | 13.93 | 44.28 | 16.34 |
|  | maker-scaffold05269-snap-gene-0.14 | ABF4 | 76.26 | 65.21 | 18.41 | 78.02 | 59.05 |
|  | augustus_masked-scaffold00073-abinit-gene-0.7 | PP2C | 0.61 | 0.46 | 1.97 | 1.42 | 2.09 |
|  | augustus_masked-scaffold13863-abinit-gene-0.0 | PP2C29 | 13.50 | 5.30 | 5.22 | 13.75 | 17.19 |
|  | maker-scaffold00024-augustus-gene-2.20 | PP2C55 | 0.14 | 0.03 | 0.66 | 0.00 | 0.08 |
|  | maker-scaffold00081-augustus-gene-1.32 | PP2C47 | 7.68 | 6.07 | 3.02 | 0.54 | 1.60 |
|  | maker-scaffold00791-snap-gene-0.38 | PP2C22 | 4.80 | 2.97 | 13.97 | 2.63 | 3.74 |
|  | maker-scaffold00859-augustus-gene-0.54 | PP2C25 | 3.70 | 0.31 | 0.66 | 1.89 | 15.95 |
|  | maker-scaffold01252-augustus-gene-0.35 | PP2C57 | 9.52 | 3.78 | 3.81 | 15.62 | 16.89 |
|  | maker-scaffold01316-augustus-gene-0.40 | PP2C25 | **91.41** | 13.23 | 11.48 | 21.24 | 43.96 |
|  | maker-scaffold01448-augustus-gene-0.20 | PP2C16 | 15.64 | 11.30 | 23.22 | 33.75 | 42.23 |
|  | maker-scaffold01727-augustus-gene-0.36 | PP2C47 | 9.29 | 6.86 | 4.37 | 1.31 | 2.65 |
|  | maker-scaffold01769-augustus-gene-0.43 | PP2C24 | 16.36 | 10.99 | 23.83 | 17.14 | 54.32 |
|  | maker-scaffold01970-snap-gene-0.42 | PP2C8 | 0.84 | 1.30 | 15.42 | 0.13 | 0.55 |
|  | maker-scaffold02122-augustus-gene-0.26 | PP2C65 | 0.80 | 7.90 | 4.77 | 0.25 | 0.14 |
|  | maker-scaffold02651-snap-gene-0.28 | PP2C44 | 8.00 | 0.95 | 0.77 | 1.07 | 0.67 |
|  | maker-scaffold06168-augustus-gene-0.19 | PP2C23 | 6.01 | 2.41 | 9.58 | 5.57 | 7.65 |
|  | maker-scaffold06866-snap-gene-0.20 | PP2C53 | 3.49 | 3.18 | 1.14 | 1.92 | 0.93 |
|  | maker-scaffold07312-augustus-gene-0.19 | PP2C72 | 12.56 | 5.25 | 3.22 | 9.50 | 8.14 |
|  | maker-scaffold07599-augustus-gene-0.6 | PP2C9 | 35.47 | 30.88 | 37.06 | **80.35** | **70.66** |
|  | maker-scaffold08423-snap-gene-0.14 | PP2C27 | 123.78 | 59.96 | 36.51 | **237.77** | **182.10** |
|  | maker-scaffold12770-snap-gene-0.6 | PP2C2 | 0.19 | 0.19 | 4.07 | 0.29 | 1.09 |
|  | maker-scaffold13625-augustus-gene-0.4 | PP2C44 | 94.58 | 52.30 | 39.66 | **154.23** | **121.78** |
|  | maker-scaffold19635-augustus-gene-0.2 | PP2C58 | 5.25 | 2.74 | 4.11 | 4.44 | 14.62 |
|  | maker-scaffold22596-augustus-gene-0.3 | PP2C72 | 10.73 | 1.01 | 1.25 | 1.41 | 0.47 |
|  | snap_masked-scaffold01945-abinit-gene-0.28 | PP2C34 | 5.70 | 4.15 | 1.76 | 0.54 | 0.90 |
|  | snap_masked-scaffold02422-abinit-gene-0.18 | PP2C75 | 8.34 | 11.56 | 43.15 | 7.38 | 11.58 |
|  | augustus_masked-scaffold00098-abinit-gene-1.6 | BZIP44 | **326.27** | 72.50 | 195.82 | 60.14 | 156.94 |
|  | augustus_masked-scaffold00355-abinit-gene-0.3 | BZIP53 | 6.47 | 3.25 | 4.43 | 1.11 | 8.43 |
|  | augustus_masked-scaffold01203-abinit-gene-0.13 | BZIP43 | 5.11 | 1.19 | 0.23 | 24.41 | 14.23 |
|  | augustus_masked-scaffold01845-abinit-gene-0.6 | BZIP53 | 117.37 | 73.56 | 89.80 | **195.05** | **198.64** |
|  | maker-scaffold01092-snap-gene-0.46 | BZIP34 | 19.84 | 3.82 | 3.88 | 3.74 | 3.86 |
|  | maker-scaffold03489-snap-gene-0.27 | BZIP9 | 16.76 | 12.06 | 37.61 | 21.37 | 77.64 |
|  | snap_masked-scaffold22009-abinit-gene-0.1 | BZIP2 | 165.55 | 6.85 | 10.02 | **132.98** | **206.37** |
|  | maker-scaffold05267-snap-gene-0.12 | DPBF3 | 157.85 | 97.48 | 99.14 | 37.02 | 58.12 |
| JA | augustus_masked-scaffold01291-abinit-gene-0.8 | MYC2 | 40.77 | 3.73 | 9.03 | 6.19 | 3.45 |
|  | augustus_masked-scaffold01491-abinit-gene-0.3 | MYC2.1 | 303.74 | 35.57 | 52.29 | 74.22 | 85.02 |
|  | augustus_masked-scaffold15592-abinit-gene-0.0 | MYC2.2 | 0.96 | 0.087 | 1.22 | 1.67 | 1.21 |
|  | maker-scaffold01036-snap-gene-0.38 | TIFY9 | 36.95 | 12.29 | 86.58 | 7.14 | 16.37 |
|  | maker-scaffold02140-snap-gene-0.24 | TIFY6B | 13.08 | 13.51 | 21.05 | 2.08 | 3.99 |
|  | maker-scaffold02210-augustus-gene-0.24 | TIFY10A | 397.35 | 43.06 | 227.08 | **228.22** | **297.97** |
|  | maker-scaffold04739-augustus-gene-0.10 | TIFY8 | 62.78 | 77.31 | 79.43 | 23.14 | 34.12 |
|  | maker-scaffold03603-snap-gene-0.25 | JMT | 2.85 | 0.97 | 0.57 | 0.44 | 0.92 |
| SA | maker-scaffold01645-augustus-gene-0.32 | PYAL1 | 113.57 | 27.97 | 213.38 | 58.78 | 249.24 |
|  | augustus_masked-scaffold01496-abinit-gene-0.2 | PYAL1.1 | 2.87 | 0.68 | 4.79 | 1.27 | 11.35 |
|  | augustus_masked-scaffold26150-abinit-gene-0.0 | PYAL | 4.51 | 2.59 | 3.91 | 8.53 | 9.24 |
|  | maker-scaffold30697-augustus-gene-0.2 | PYAL.1 | 31.96 | 14.02 | 51.48 | 44.97 | 77.91 |
|  | augustus_masked-scaffold09836-abinit-gene-0.0 | PYAL2 | 36.19 | 19.37 | 58.81 | 52.15 | 109.13 |
|  | augustus_masked-scaffold20658-abinit-gene-0.0 | PYAL2.1 | 4.41 | 2.15 | 2.34 | 9.03 | 9.34 |
|  | augustus_masked-scaffold09151-abinit-gene-0.4 | SABP2 | 4.06 | 1.61 | 0 | 6.39 | 4.81 |
|  | maker-scaffold17654-augustus-gene-0.3 | SABP2.1 | 189.16 | 110.06 | 83.15 | 492.20 | 436.86 |
